# MOVICShiny: An interactive website for multi‐omics integration and visualisation in cancer subtyping

**DOI:** 10.1002/ctm2.1606

**Published:** 2024-03-06

**Authors:** Junkai Zhu, Yuyao Zhu, Xin Wang, Wenxuan Cheng, Shuaiyi Wang, Junluo Yang, Wenxuan Wang, Yuhang Wang, Jialin Meng, Xiaofan Lu, Fangrong Yan

**Affiliations:** ^1^ Research Center of Biostatistics and Computational Pharmacy China Pharmaceutical University Nanjing People's Republic of China; ^2^ Department of Urology The First Affiliated Hospital of Anhui Medical University Institute of Urology Anhui Province Key Laboratory of Urological and Andrological Diseases Research and Medical Transformation Anhui Medical University Hefei People's Republic of China; ^3^ Department of Cancer and Functional Genomics Institute of Genetics and Molecular and Cellular Biology CNRS/INSERM/UNISTRA Illkirch France

Dear Editor,

Cancer subtyping holds significant importance in tumour research, yet researchers lacking programming skills face challenges with R packages. To address this challenge, we develop MOVICShiny, an interactive website designed for cancer subtyping through multi‐omics integration and visualisation for coding‐free multi‐omics analysis based on MOVICS R package. Our software has subsequently been deployed on a cloud server, providing an efficient platform for conducting multi‐omics analysis on diverse cancer types reliably and accurately. The entire pipeline is illustrated in Figure 1.

**FIGURE 1:**
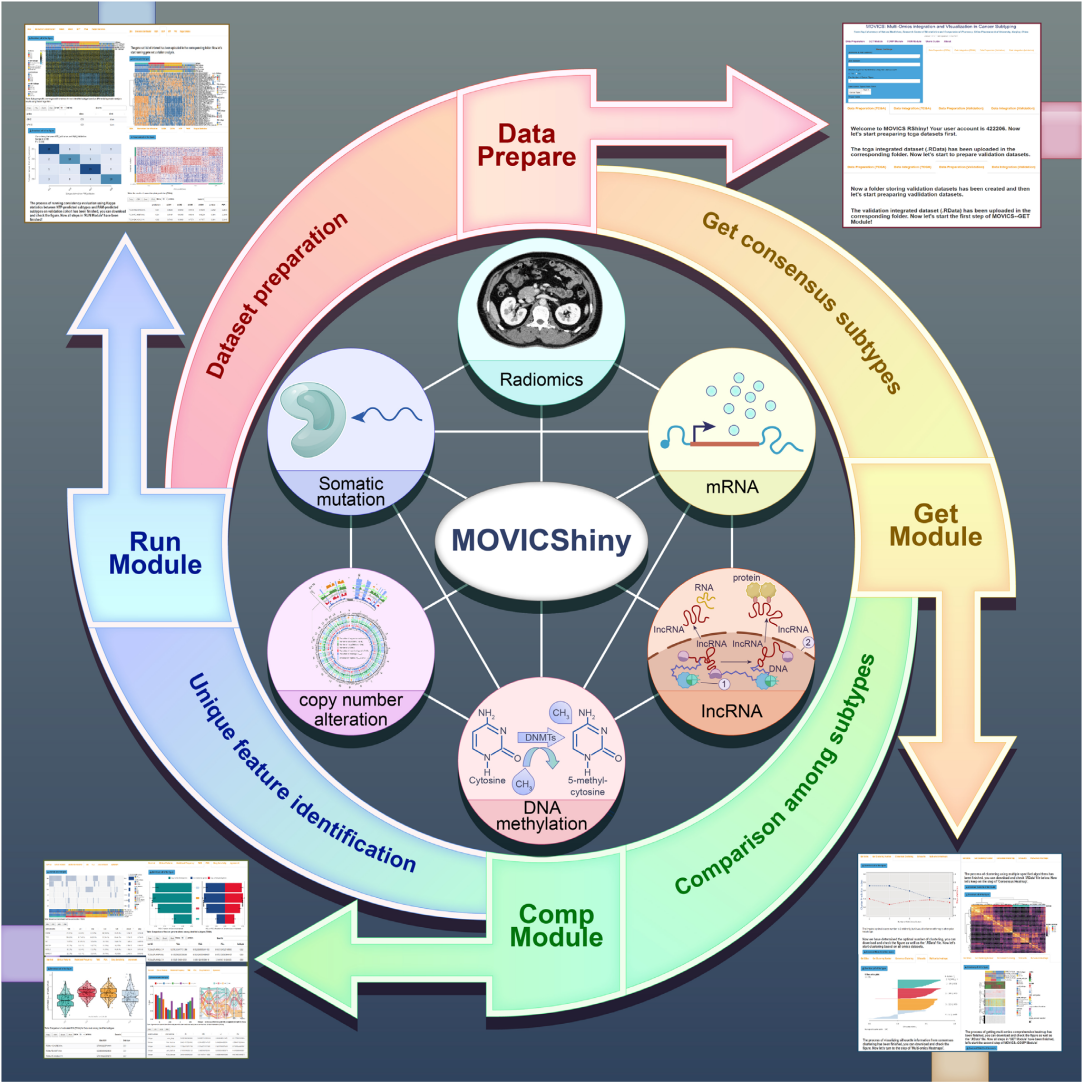
Schematic view of MOVICShiny pipeline of four modules. These modules follow a sequential order and are designed to facilitate seamless data analysis. The inner circles represent six types of omics data, the outer circles represent four modules of the website and the arrows show partial result display of each module.

Cancer subtyping is pivotal in guiding personalised treatments and identifying potential therapeutic targets. Researchers increasingly favour multi‐omics analysis, integrating diverse data types, over traditional single‐omics approaches.^1^ This approach provides comprehensive insights into disease mechanisms, uncovering heterogeneity among subtypes from various angles. This widely used multi‐omics analysis MOVICS R package not only combines different omics data such as transcriptome, DNA methylation, copy number alterations and somatic mutation for multi‐algorithm consensus clustering, but also includes subtype comparisons such as survival analysis, clinical variables correlation analysis, somatic mutation analysis, copy number alterations analysis, and drug sensitivity analysis, as well as downstream analyses such as differential expression analysis, pathway analysis and external validation. Although MOVICS R package is extensively utilised in various cancers, including bladder, prostate and lung adenocarcinoma, its usage demands proficiency in R programming and meticulous parameter adjustments. More importantly, data for analysis should be well prepared by researchers. Thus, integrating codes for multi‐omics analysis and data preparation into user‐friendly software becomes imperative.

To ensure real‐time result visualisation, our software incorporates three areas. The upper area includes the title and module switching options. The lower area is further divided into two parts, with parameters settings on the left and results display on the right. Our software is divided into four modules, including ‘Data Preparation’, ‘GET Module’, ‘COMP Module’ and ‘RUN Module’.

‘Data Preparation’ is divided into The Cancer Genome Atlas (TCGA) dataset preparation and external validation dataset preparation. For TCGA, options include direct acquisition from internal datasets, automatic websites download, specific URLs download, local files upload and combined ‘.RData’ files upload. External validation datasets rule out direct and automatic options due to diverse sources.

‘GET Module’ is divided into five submodules. ‘Get Elites’ processes omics data sequentially, performing dimensionality reduction based on predefined rules for subsequent cluster analysis. ‘Get Optimal Clustering Number’ suggests the optimal clustering number. ‘Consensus Clustering’ offers 10 algorithms (e.g. iClusterBayes, SNF, PINSPlus) for user‐selected clustering methods. At least two algorithms are needed for consensus clustering. ‘Silhouette’ calculates and visualises sample similarity within each subtype using Silhouette Coefficient.^2^ ‘Multi‐omics Heatmaps’ integrates omics data and clustering results to visualise subtype expression differences.^3^

‘COMP Module’ analyses subtypes form clustering, comprising seven submodules. ‘Compare survival outcome’ compares survival variances among subtypes. ‘Compare clinical features’ identifies clinical variables linked to subtypes. ‘Compare mutational frequency’ compares mutations for specific genes under certain criteria, highlighting significant subtype differences. ‘Compare total mutation burden’ compares the total mutation burden (TMB) among each subtype.^4^ ‘Compare fraction genome altered’ assesses the variations in fraction of genome altered (FGA), fraction of genome gained (FGG) and fraction of genome lost (FGL)^5^ across different subtypes. ‘Compare drug sensitivity’ uses IC_50_ to compare drug responses from Genomics of Drug Sensitivity in Cancer (GDSC)^6^ among subtypes. ‘Compare agreement with other subtypes’ compares clustering consistency with existing classification (e.g. PAM50, pStage).

‘RUN Module’ performs downstream analyses, divided into seven submodules. ‘Run differential expression analysis’ utilises DESeq2, edgeR and limma algorithms to highlight differentially expressed genes specific to each subtype. For each subtype, ‘Run biomarker identification procedure’ sets conditions to further screen out significantly upregulated and down‐regulated marker genes. Then ‘Run gene set enrichment analysis’ identifies pathways and calculates enrichment scores^7^ of each subtype. Analogously, ‘Run gene set variation analysis’ calculates enrichment scores^7^ of interest for each sample. ‘Run nearest template prediction’ utilises the Nearest Template Prediction (NTP)^8^ method to predict external dataset subtypes using marker genes and assesses consistency. ‘Run partition around medoids classifier’ predicts external dataset subtypes with Partition Around Medoids (PAM)^9^ and evaluates consistency via in‐group proportion (IGP).^10^ Results from NTP and PAM methods validate clustering in ‘COMP Module’. ‘Run consistency evaluation using Kappa statistics' assesses clustering and prediction consistency.

In our software development, we have extended radiomics features extracted from raw images on the basis of original data types from MOVICS, containing mRNA, lncRNA, DNA methylation, copy number alterations and somatic mutation. With the help of our software, parameters can be adjusted using user‐friendly options or input fields, followed by a simple click to obtain data and results. Our software promptly presents the acquired results in visual or tabular format, facilitating easy monitoring and adjustments. It also offers a convenient download feature for users to retrieve data and results. Moreover, our software is deployed on a high‐performance cloud server that meets the computing and storage requirements for data preparation and multi‐omics analysis.

Our software underwent rigorous testing across its entire development process. Web page testing ensures alignment between the developed web pages and the design, checking for missing parameters and the accuracy of option settings, among other aspects. Logic testing involved comparing data and results related to bladder cancer between our software and R package to ensure they met expectations. Besides, logic testing also checks whether our software can normally respond to user operations. Details of testing data and results are shown in Supporting Information.

Despite the achievements in software development, there are several areas that require further improvement. First, refining our software's aesthetics and user interface is essential. Second, optimising the methods employed in our software to enhance model efficiency represents a future research direction. Moreover, we are actively considering the inclusion of additional data types, specifically proteomics and metabolomics.

In conclusion, we developed a shiny network analysis software for multi‐omics based on MOVICS R package. Our software has undergone thorough testing and is now in use, offering researchers with limited programming skills the ability to perform comprehensive tumour multi‐omics analysis. Notably, our software outperforms the MOVICS R package with improved readability, user experience, research efficiency, and response speed.

## AUTHOR CONTRIBUTIONS

Conceptualisation: Junkai Zhu, Yuyao Zhu, Jialin Meng and Xiaofan Lu. Software: Junkai Zhu, Yuyao Zhu and Xin Wang. Methodology: Wenxuan Cheng, Jialin Meng, Xiaofan Lu and Fangrong Yan. Formal analysis: Junluo Yang. Investigation: Wenxuan Wang and Yuhang Wang. Data curation: Shuaiyi Wang. Writing original draft: Junkai Zhu, Yuyao Zhu, Xin Wang and Wenxuan Cheng. Writing review and editing: Jialin Meng, Xiaofan Lu and Fangrong Yan. Funding acquisition: Fangrong Yan. Supervision: Fangrong Yan.

## FUNDING

This work was supported by the National Natural Science Foundation of China (No.81973145, No.82273735) and Key R&D Program of Jiangsu Province (Social Development) (BE2020694).

## CONFLICT OF INTEREST STATEMENT

The authors declare that they have no conflict of interest.

## ETHICS STATEMENT

As the data used in this study are publicly available, no ethical approval is required.

Additionally, our platform is currently free to use, and we have no charging plans for a short period of time. Before using the platform, you should first write an application email containing name, affiliation, application purpose, etc. to xlu.cpu@foxmail.com to apply for a username and a user account. After that, you can log in the platform with the email used for application as Username and the assigned account as User Account. Then you can specify the required parameters to start a new analysis or continue an existing one.

## Supporting information



Additional supporting information may be found in the online version of the article at the publisher's website.

## Data Availability

Our web server is freely accessible at http://www.movics‐cpu.com:3838/. The source code, test data, Users Guide and About are available on GitHub at https://github.com/xlucpu/MOVICShiny. Our tutorial video is available on YouTube at the following link: https://youtu.be/QrzU2VZHl3A. This provides users with a more intuitive and easily understandable guide to use the software. According to the tutorial video, Users Guide and test data, our software can be easily utilised. It is worth noting that ‘blca.tcga.RData’ represents the TCGA dataset, while the other two serve as validation datasets, which should be uploaded after another has been validated.
