# Supplementary material for: MOVICShiny: An interactive website for multi‐omics integration and visualisation in cancer subtyping
Source: Clin Transl Med. 2024 Mar 6;14(3):e1606. doi: 10.1002/ctm2.1606 (PMC10915727; doi:10.1002/ctm2.1606)
Supplement: Supplementary file 1 — Additional supporting information may be found in the online version of the article at the publisher's website. [file CTM2-14-e1606-s001.docx]

**MOVICShiny: An interactive website for multi-omics integration and visualization in cancer subtyping**

**Supporting Information**

**Contents**

[**1 Test data of bladder cancer 1**](#_Toc159700017)

[**2 Display of software and testing results 1**](#_Toc159700018)

[**2.1 Result display for “Data Preparation” 2**](#_Toc159700019)

[**2.2 Result display for “GET Module” 2**](#_Toc159700020)

[**2.3 Result display for “COMP Module” 3**](#_Toc159700021)

[**2.4 Result display for “RUN Module” 4**](#_Toc159700022)

[**2.5 Result display for external validation 6**](#_Toc159700023)

[**3 Supplementary figures 7**](#_Toc159700024)

[**4 References 51**](#_Toc159700025)

# Test data of bladder cancer

We have extracted 396 common samples from five omics data types, including mRNA (tpm values), lncRNA (tpm values), DNA methylation, copy number alterations, and somatic mutation from TCGA database in “Data Preparation”. Before data cleaning and dimensionality reduction, mRNA dataset has 19469 features, lncRNA dataset has 13060 features, DNA methylation dataset has 10871 features, copy number alterations dataset has 48210 features, and somatic mutation dataset has 16824 features. After that, we extracted top 1500 highly variable features for mRNA dataset, lncRNA dataset, DNA methylation dataset, and copy number alterations dataset respectively at the step of “Get Elites” in “GET Module”. For somatic mutation dataset, we extracted 1358 features whose mutation frequency divided by sample size is higher than 0.03. In addition, we also collected the information of gene expression (count values), copy number segment, Minor allele frequency (MAF), as well as clinical and survival.

For external validation, we obtained two cohorts named “affy” and “illumina” in “Data Preparation”, with 117 samples and 286 samples separately. Both these two cohorts only contain mRNA as well as clinical and survival information. After personalized processing, we extracted 20760 and 15906 features for mRNA dataset in two cohorts respectively.

# Display of software and testing results

Up to now, the software has been developed and has passed all the tests. It has been deployed on cloud server for multi-omics analysis of different cancers (Figure S1-S6). The access address is <http://www.movics-cpu.com:3838/>. In addition, the software was tested on bladder cancer, and the obtained data and results are the same as MOVICS R package, which will be shown below by module.

## Result display for “Data Preparation”

The software will give feedback to the users after finishing the process of preparing TCGA dataset and validation dataset (Figure S7, S8).

## Result display for “GET Module”

At the step of “Get Elites”, the software will give feedback to the users after finishing the process of “Get Elites” on TCGA dataset and validation dataset (Figure S9, S10). After that, the software will plot according to the values of CPI and Gaps-statistics and suggest the optimal clustering number when the sum of CPI and Gaps-statistics is the largest at the step of “Get Clustering Number”. However, we should also consider the prior information when determining the optimal clustering number, which will be used for the following “Consensus Clustering” step. Here we combine prior information to determine “4” as the optimal clustering number (Figure S11). Then, the software will give feedback to the users if the “Consensus Clustering” step is finished (Figure S12). Moreover, a consensus heatmap will be also displayed to reflect the quality of consensus clustering results (Figure S12). Furthermore, to evaluate the similarity between samples in each subtype derived from clustering results, the software calculates and visualizes the Silhouette Coefficient to evaluate the clustering results at the step of “Silhouette” (Figure S13). Silhouette Coefficient ranges from -1 to 1, and the larger value indicates better clustering results. Additionally, the step of “Multi-omics Heatmaps” generates a multi-omics heatmap for users to observe the expression differences of features in each omics data for each subtype (Figure S14). Because the generated multi-omics heatmap is relatively large, it may not be displayed clearly enough on the website. So, users can download the heatmap, and then enlarge it for observation.

## Result display for “COMP Module”

First, the software will generate Kaplan-Meier curve ^1^ for each subtype at the step of “Compare survival outcome”, and compare the survival differences among each subtype ^2^ at the same time (Figure S15). Overall *p* value that is less than 0.05 reveals significant survival differences among each subtype. Besides, the software will generate a table to display the comparison results of clinical features among subtypes at the step of “Compare clinical features” (Figure S16). If *p* value is less than 0.05, it reveals that the clinical feature is significantly correlated with subtypes. At the step of “Compare mutational frequency”, the software will generate a table to show the comparison of mutations among subtypes for specific genes with mutation frequency that is higher than the setting cutoff of 0.03 (Figure S17). Additionally, the software also generates an oncoprint to display the mutations of the genes whose mutation frequency is significantly different among subtypes on the basis of the table (Figure S17). Furthermore, the software will also generate a box-violin plot to show the comparison results of TMB among subtypes at the step of “Compare total mutation burden”, and the TMB of each sample will be also displayed through a table (Figure S18). *P* value that is less than 0.05 shows significantly different on TMB among subtypes. Then, at the step of “Compare fraction genome altered”, the software will generate bar plots to show the comparison results of FGA, FGG and FGL among subtypes, and the values of FGA, FGG, and FGL of each sample will be also displayed through a table (Figure S19). The sign of “*” reveals significantly different on FGA, or FGG, or FGL among subtypes. After that, the software will generate box-violin plots to show the IC_50_ comparison results among subtypes for Cetuximab and Erlotinib, and the estimated IC_50_ of each sample for drugs will be also displayed through tables at the step of “Compare drug sensitivity” (Figure S20, S21). *P* value that is less than 0.05 reveals significance of differences on the response to drugs among subtypes. Finally, at the step of “Compare agreement with other subtypes”, the software will generate an alluvial diagram to compare agreement between obtained subtypes and other traditional subtypes (Figure S22). In addition, a bar plot and a table are also generated to display the values of four statistical indicators which are utilized to evaluate the agreement (Figure S22), including Rand Index (RI) ^3^, Adjusted Mutual Information (AMI) ^4^, Jaccard Index (JI) ^5^, and Fowlkes-Mallows (FM) ^6^. All these indicators range from 0 to 1, and the larger the values are, the clustering results are more similar to the current classification results.

## Result display for “RUN Module”

The software will first generate a table to display the results of differential expression analysis at the step of “Run differential expression analysis” (Figure S23). Then, the software will generate heatmaps at the step of “Run biomarker identification procedure” to show the expression of the screening up-regulated and down-regulated markers in each subtype (nominal *p* value < 0.05 & adjusted *p* value < 0.05). In addition, the screening markers for each subtype are also displayed through tables (Figure S24, S25). After that, at the step of “Run gene set enrichment analysis”, the software will generate heatmaps (Figure S26, S27) to show the enrichment scores of the screening up-regulated and down-regulated pathways for each subtype (nominal *p* value < 0.05 & adjusted *p* value < 0.25). Moreover, the gene set enrichment analysis results as well as enrichment scores of the screening up-regulated and down-regulated pathways for each subtype are also displayed through tables (Figure S28-S31). Furthermore, the software will generate a heatmap to show the enrichment scores of the pathways of interest for each sample at the step of “Run gene set variation analysis” (Figure S32). Besides, the raw enrichment scores as well as the z-scored enrichment scores of the pathways of interest for each sample are also displayed through tables (S33, S34). At the step of “Run nearest template prediction”, the software will generate tables to show the subtype prediction results of each sample in TCGA dataset as well as two validation datasets through NTP. Additionally, consistency between prediction results and templates in TCGA dataset as well as two validation datasets will be evaluated through heatmaps, and higher degree of consistency indicates better prediction performance (Figure S35). At the step of “Run partition around medoids classifier”, the software will generate tables to show the subtype prediction results of each sample in TCGA dataset as well as two validation datasets using PAM. Besides, the values of IGP statistics for each subtype will be also displayed through tables, which reflects the similarity and reproducibility between the training data and testing data (Figure S36). Larger value of IGP indicates higher consistency. Here, the “training data” refers to TCGA dataset. If we want to predict the subtype of each sample in TCGA dataset, the “testing data” refers to TCGA dataset. If we want to predict the subtype of each sample in validation dataset, the “testing data” refers to validation dataset. Finally, the software will calculate Kappa statistics ^7^, and then generate heatmaps to evaluate the consistency between clustering results and prediction results or the consistency between prediction results derived from NTP and PAM method (Figure S37-S41). The greater the Kappa statistic is, the higher the degree of consistency is. Generally speaking, Kappa statistic that is greater than 0.4 indicates a high degree of consistency, and greater than 0.7 indicates an extremely high degree of consistency.

## Result display for external validation

In order to further validate the accuracy and reliability of the clustering results, we also complete partial analyses in “COMP Module” on two external validation datasets in turn. Since the two validation datasets only include mRNA as well as clinical and survival data, we only finish “Compare survival outcome”, “Compare clinical features”, “Compare drug sensitivity” and “Compare agreement with other subtypes”.

Like the TCGA dataset, the software will also generate Kaplan-Meier curve for each subtype derived from NTP and PAM methods for two validation datasets at the step of “Compare survival outcome”, and compare the survival differences among each subtype at the same time (Figure S42-S45). Besides, at the step of “Compare clinical features”, the software will also generate tables to display the comparison results of clinical features among subtypes derived from NTP and PAM methods for two validation datasets (Figure S46-S49). After that, the software will also generate box-violin plots to show the IC_50_ comparison results for drugs among subtypes derived from NTP and PAM methods for two validation datasets at the step of “Compare drug sensitivity”, and the estimated IC_50_ of each sample in two validation datasets for drugs will be also displayed through tables (Figure S50-S57). Finally, at the step of “Compare agreement with other subtypes”, the software will also generate alluvial diagrams to compare agreement between subtypes derived from NTP as well as PAM methods and other traditional subtypes for two validation datasets (Figure S58-S61). Additionally, bar plots and tables are also generated for two validation datasets to display the values of four statistical indicators which are utilized to evaluate the agreement (Figure S58-S61).

# Supplementary figures


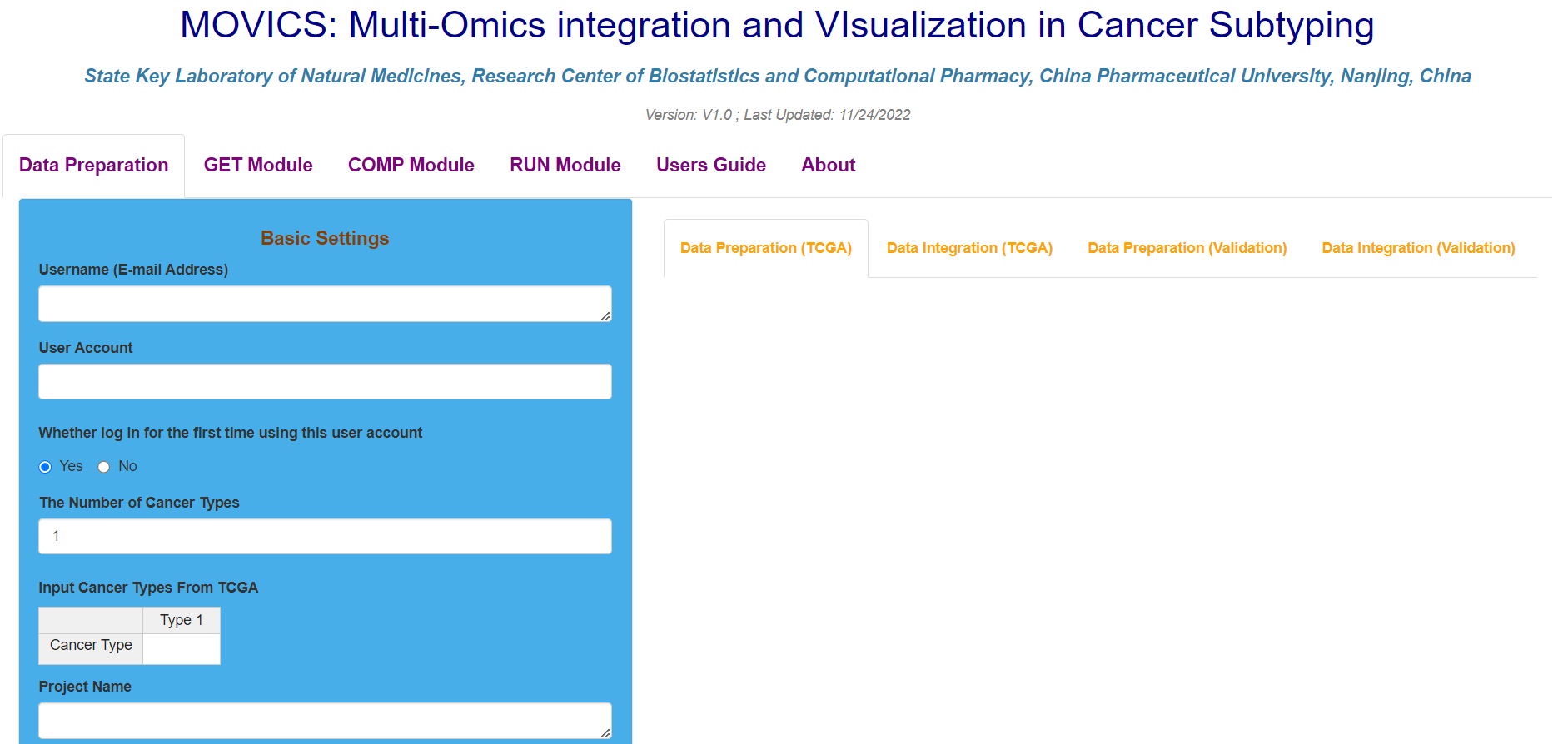


**Figure S1.** Interface display for “Data Preparation” of MOVICShiny software.


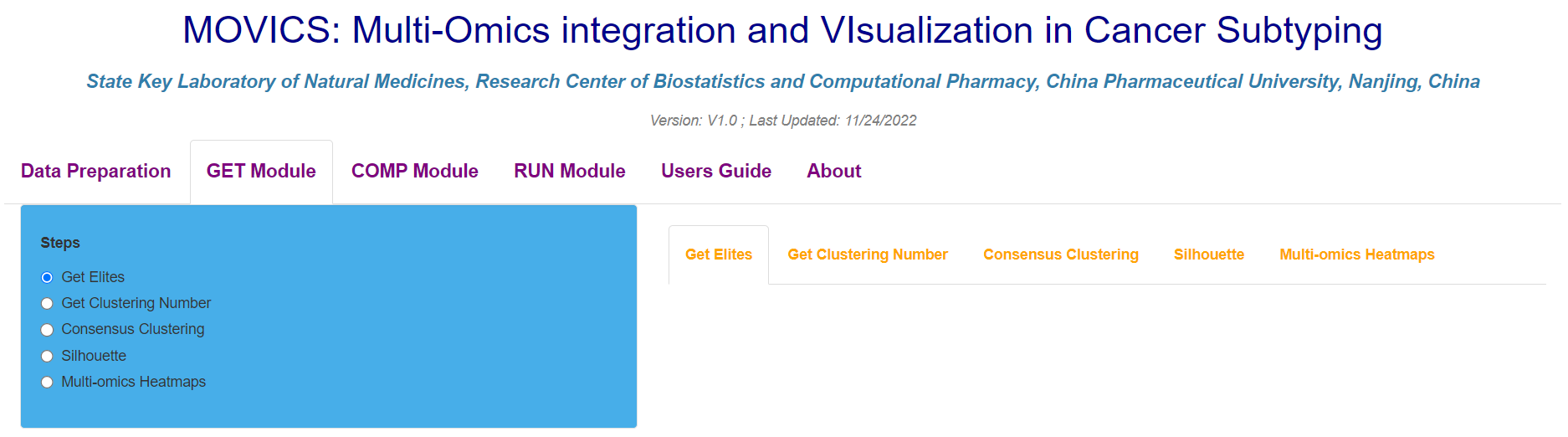


**Figure S2.** Interface display for “GET Module” of MOVICShiny software.


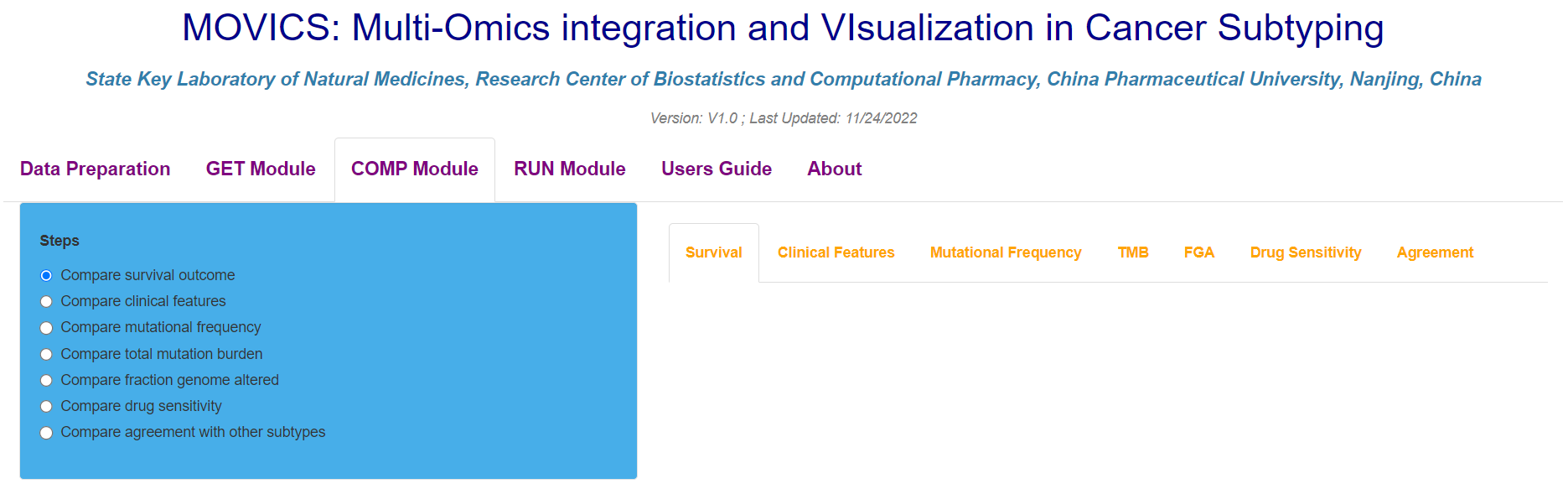


**Figure S3.** Interface display for “COMP Module” of MOVICShiny software.


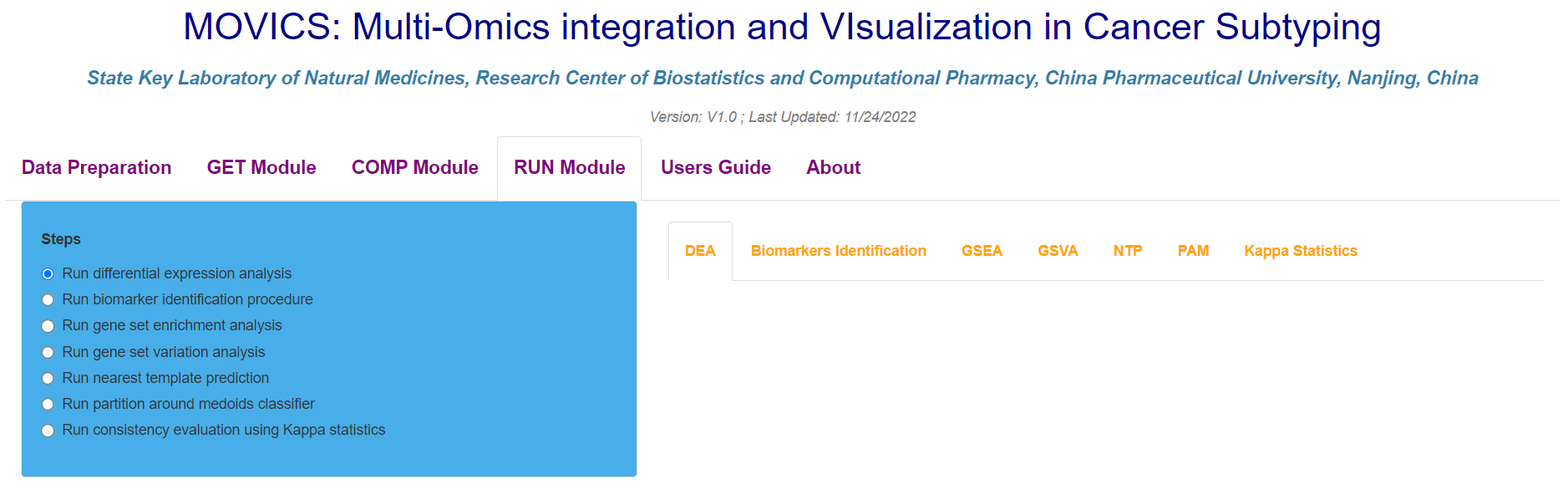


**Figure S4.** Interface display for “RUN Module” of MOVICShiny software.


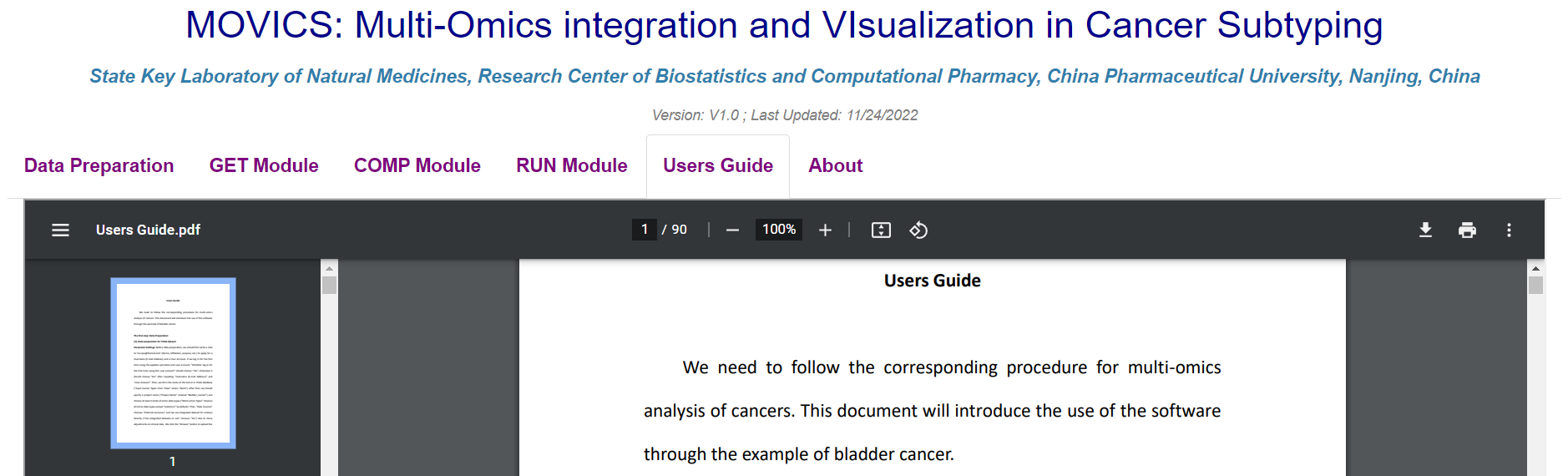


**Figure S5.** Interface display for “Users Guide” of MOVICShiny software.


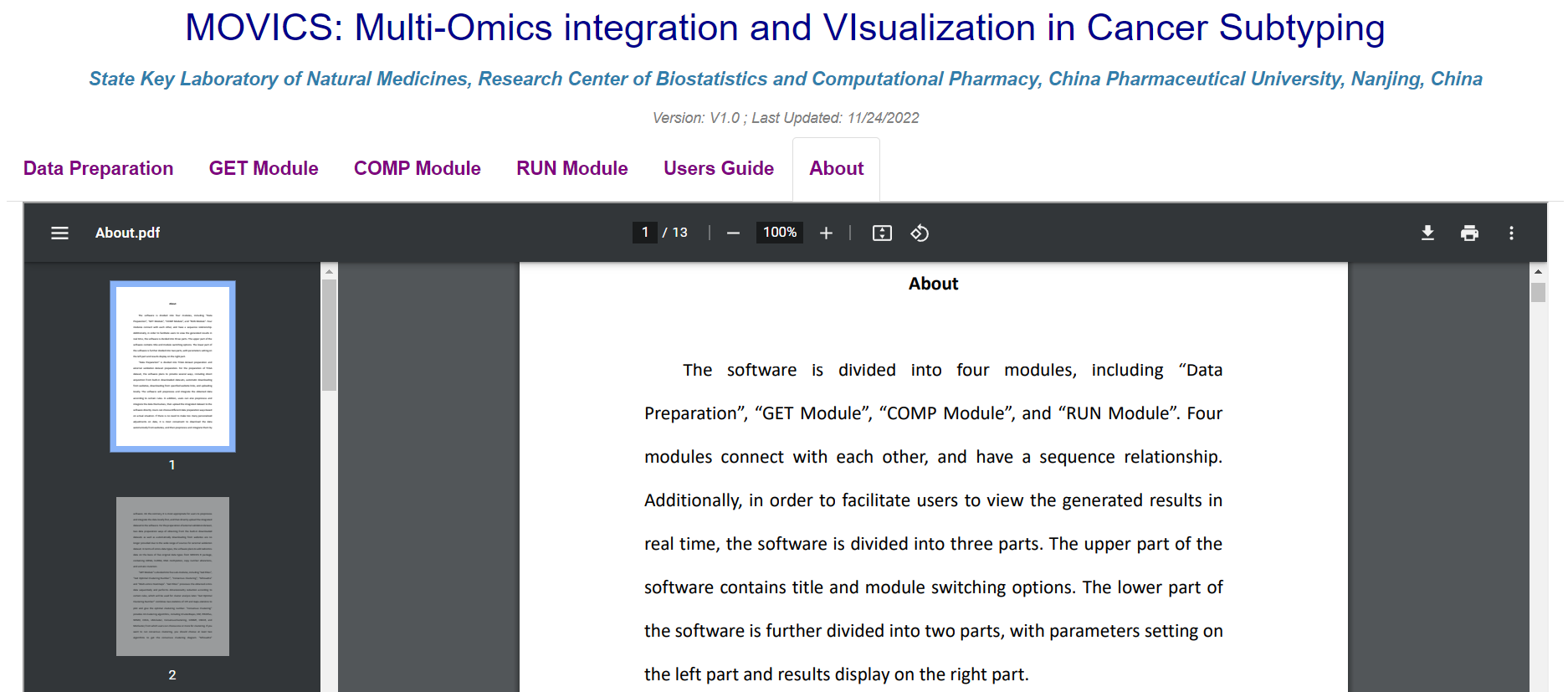


**Figure S6.** Interface display for “About” of MOVICShiny software.


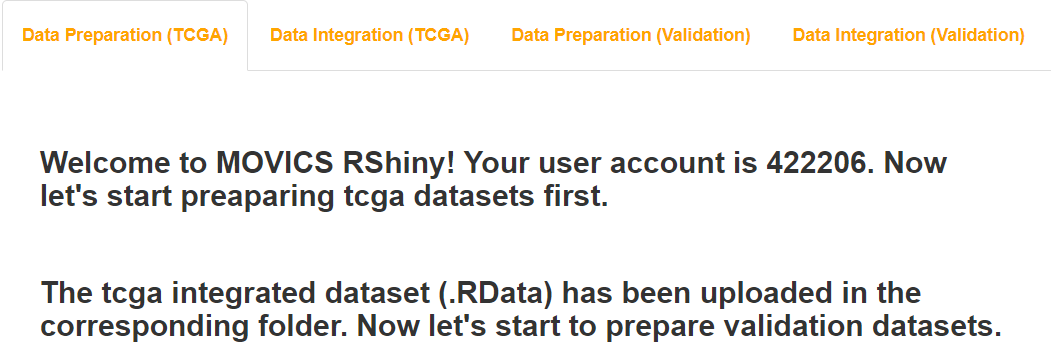


**Figure S7.** Feedback after data preparation of TCGA dataset.


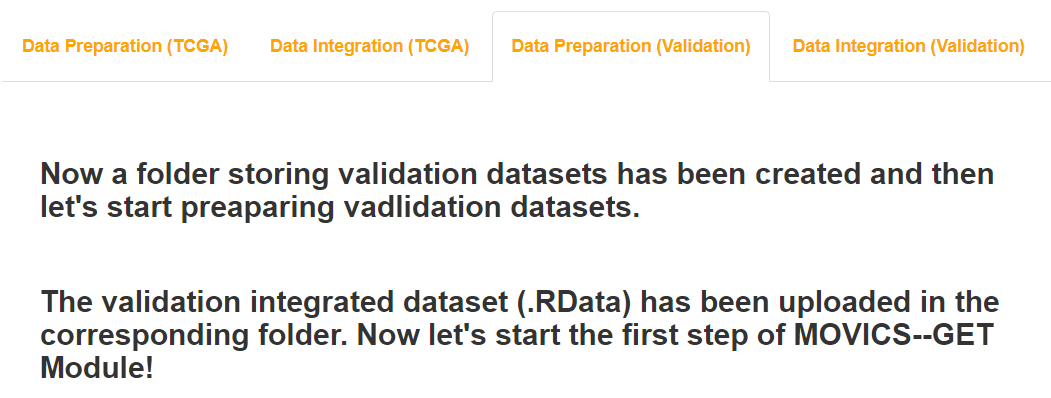


**Figure S8.** Feedback after data preparation of validation datasets.


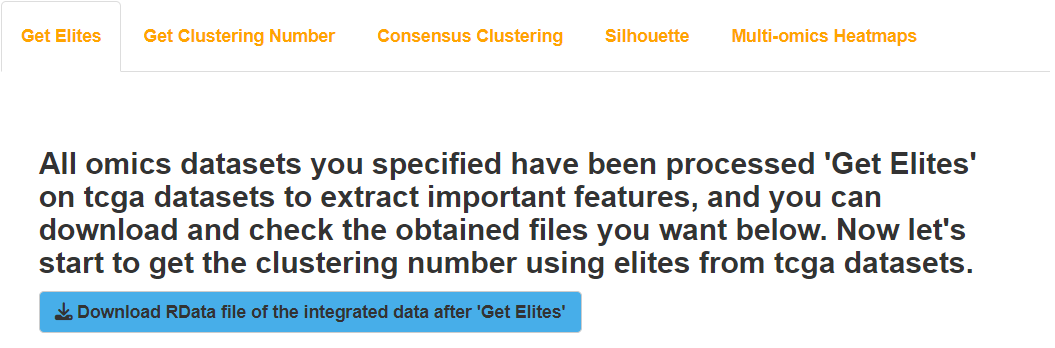


**Figure S9.** Feedback after the step of “Get Elites” in “GET Module” on TCGA dataset.


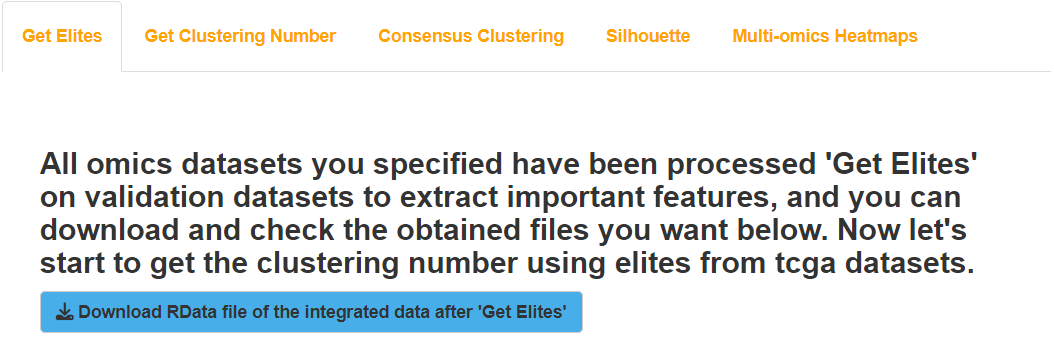


**Figure S10.** Feedback after the step of “Get Elites” in “GET Module” on validation datasets.


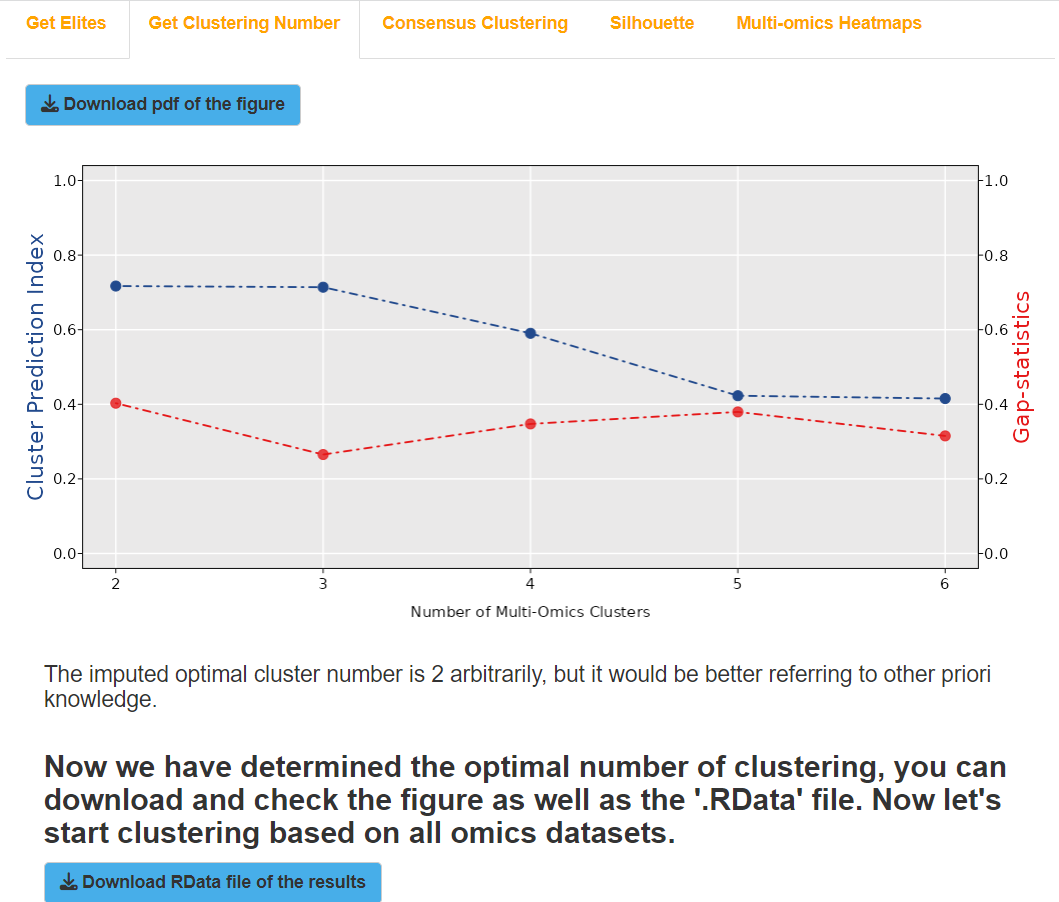


**Figure S11.** Determine the optimal clustering number for TCGA dataset.


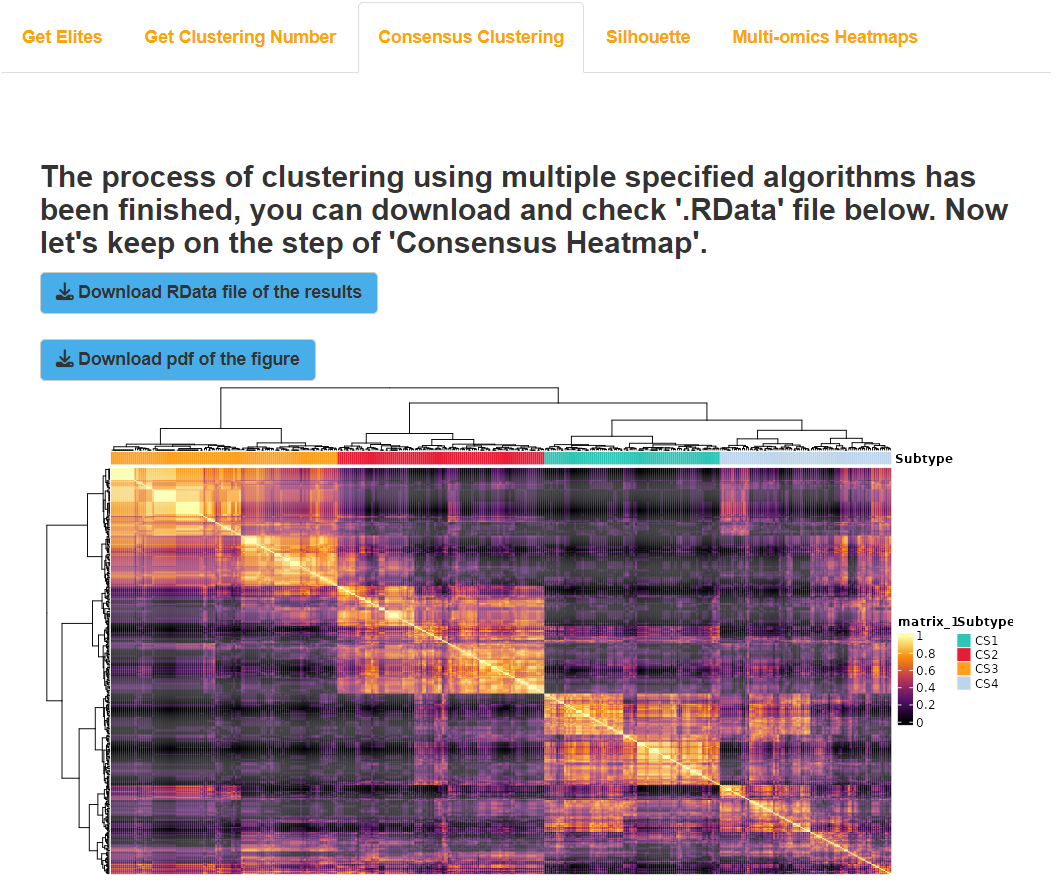


**Figure S12.** Feedback after consensus clustering with a consensus heatmap for TCGA dataset.


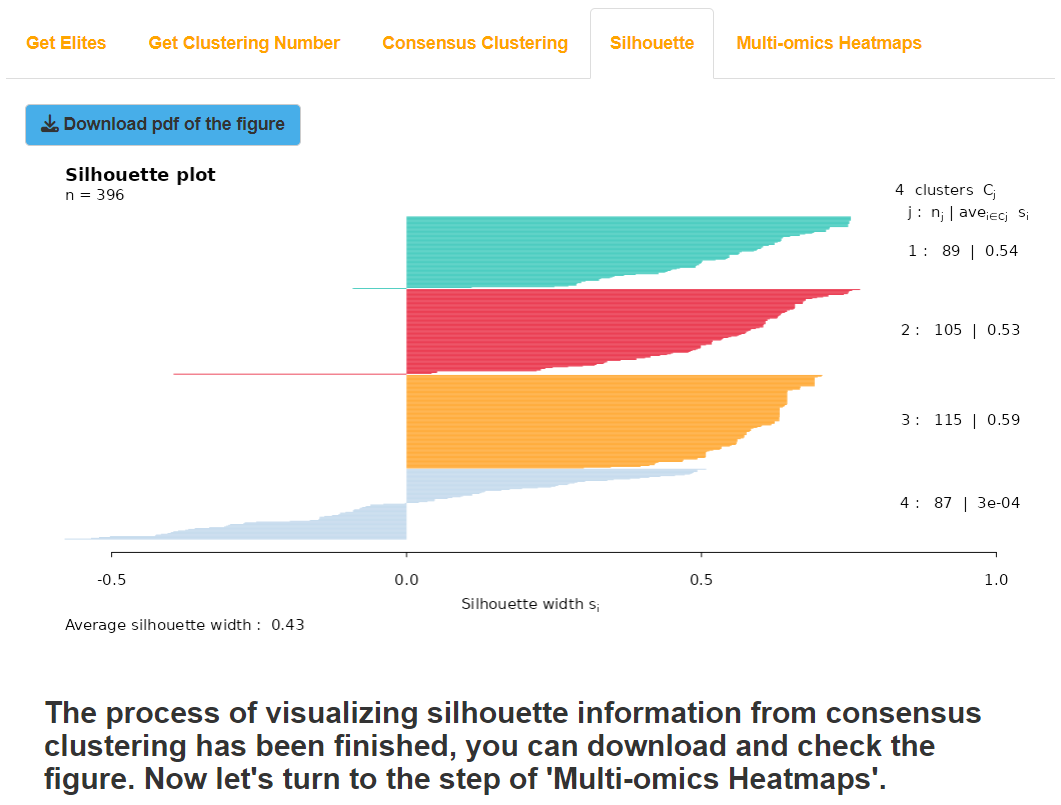


**Figure S13.** Visualization of Silhouette Coefficient to evaluate the similarity between samples in each subtype for TCGA dataset.


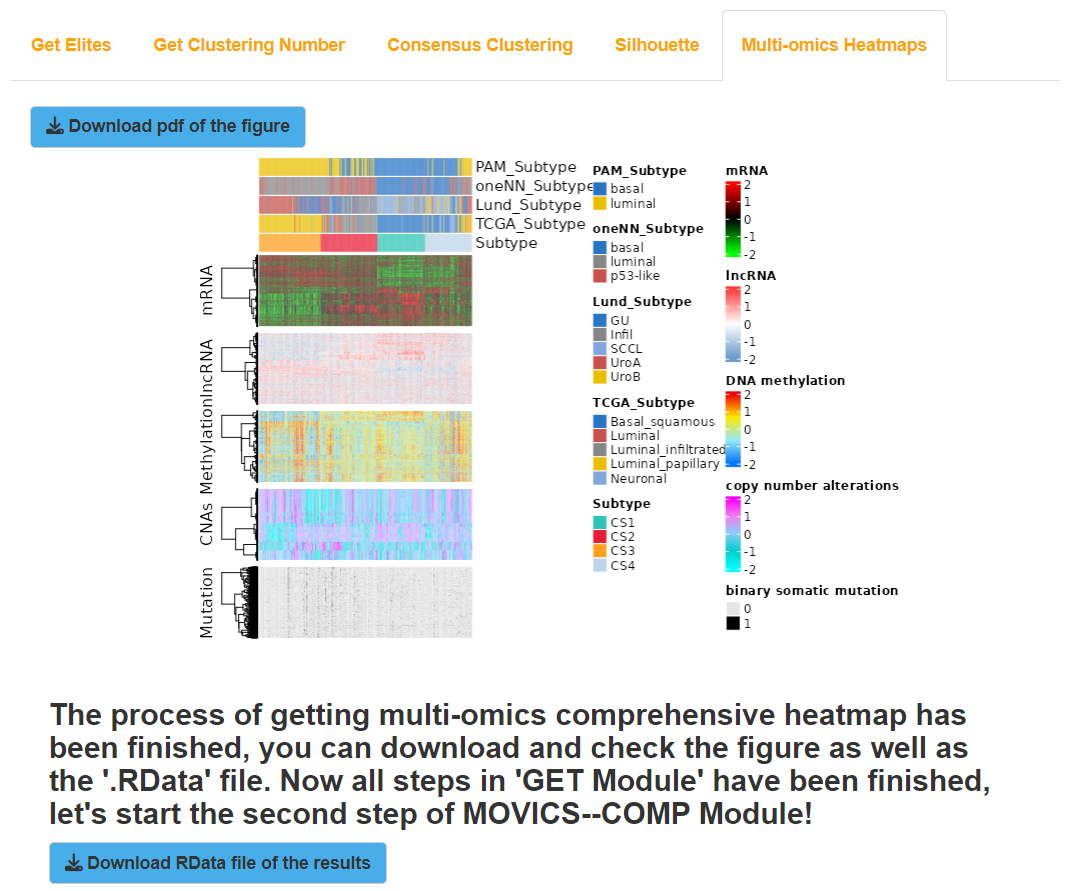


**Figure S14.** A multi-omics heatmap for TCGA dataset to observe the expression differences of features in each omics data for each subtype. Abbreviations: TCGA, The Cancer Genome Atlas.


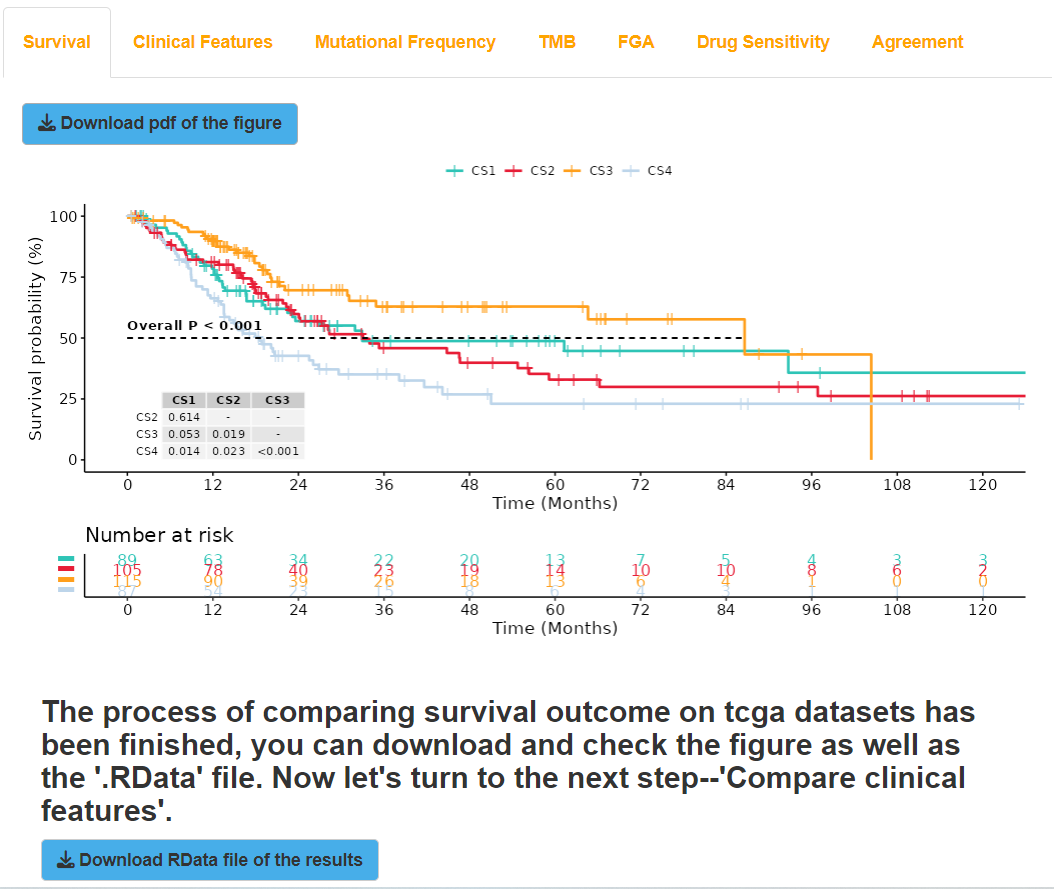


**Figure S15.** Compare the survival differences among subtypes for TCGA dataset.


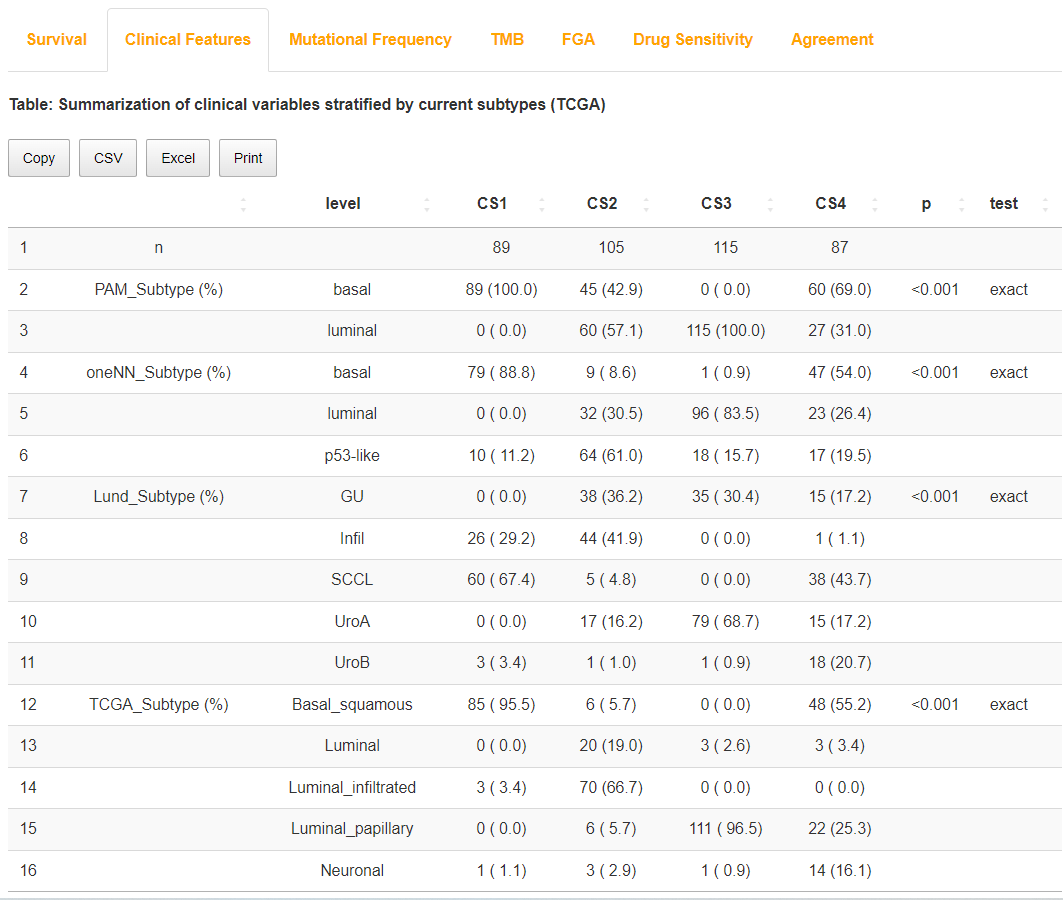


**Figure S16**. Compare the clinical features among subtypes to find out the clinical features which are significantly correlated with subtypes for TCGA dataset.


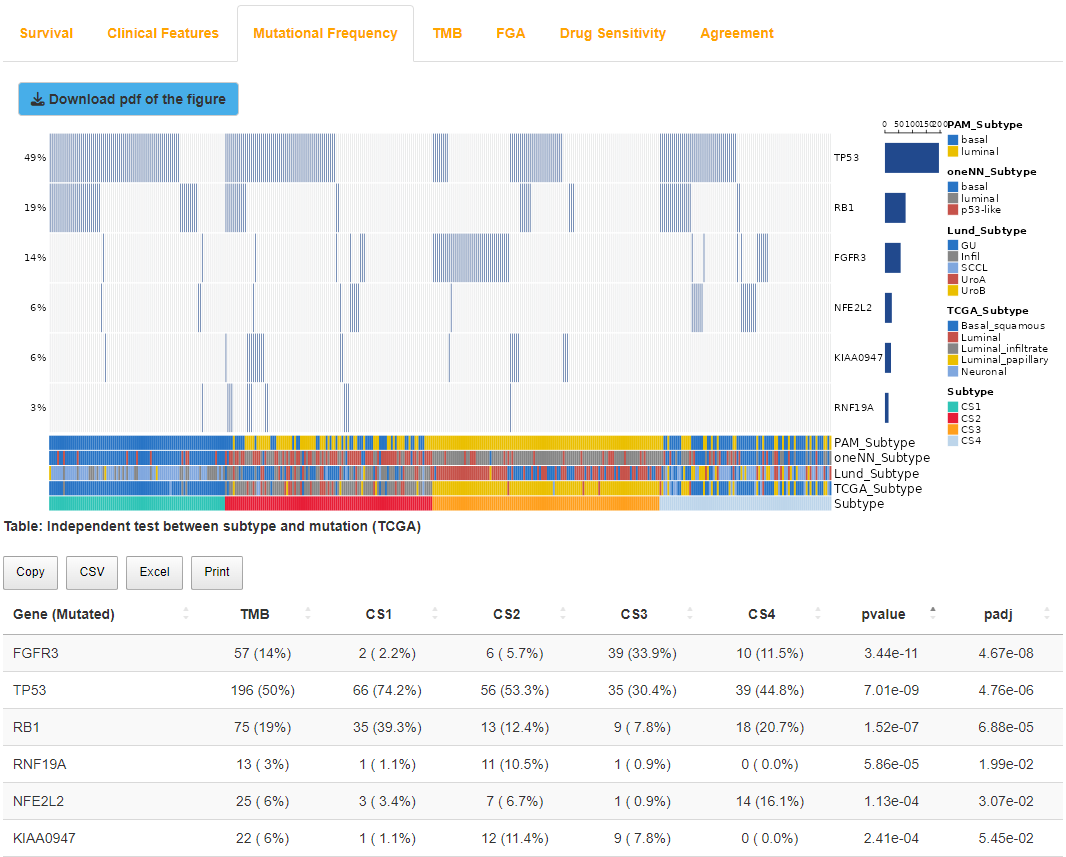


**Figure S17.** Compare the mutations among subtypes for TCGA dataset.


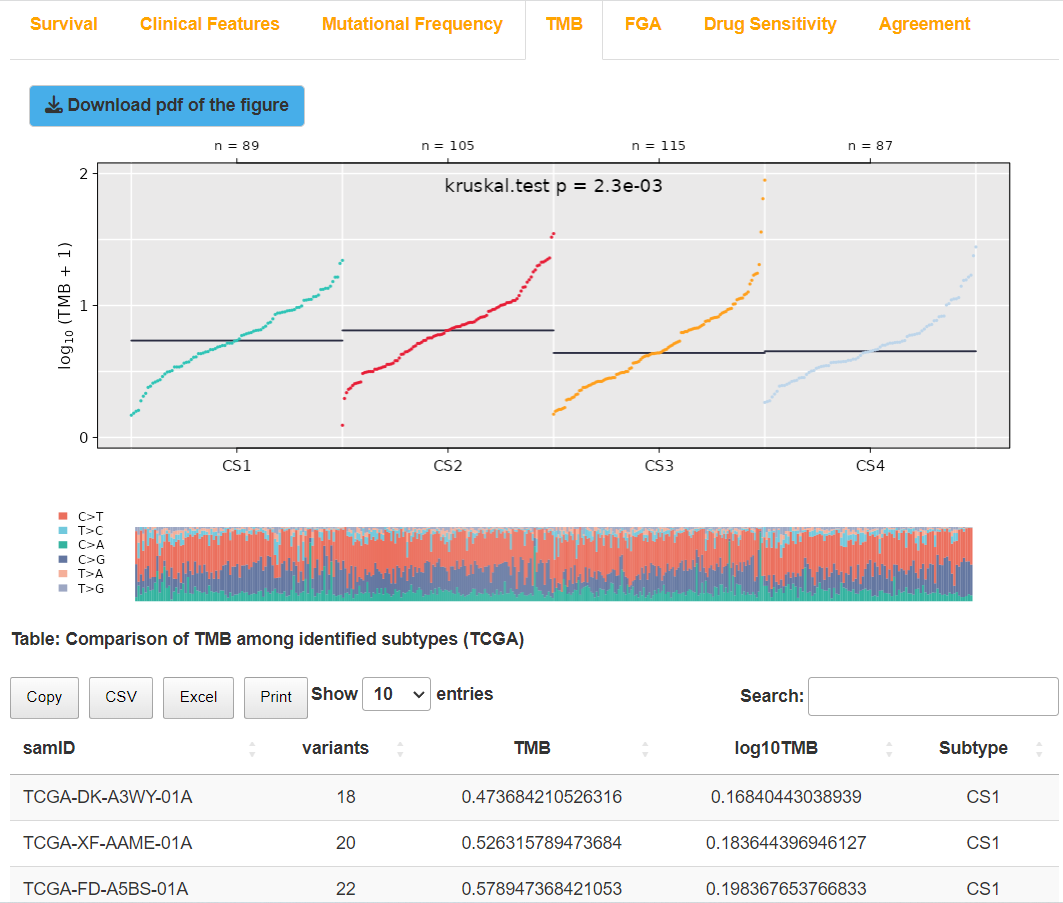


**Figure S18.** Compare the TMB among subtypes for TCGA dataset.


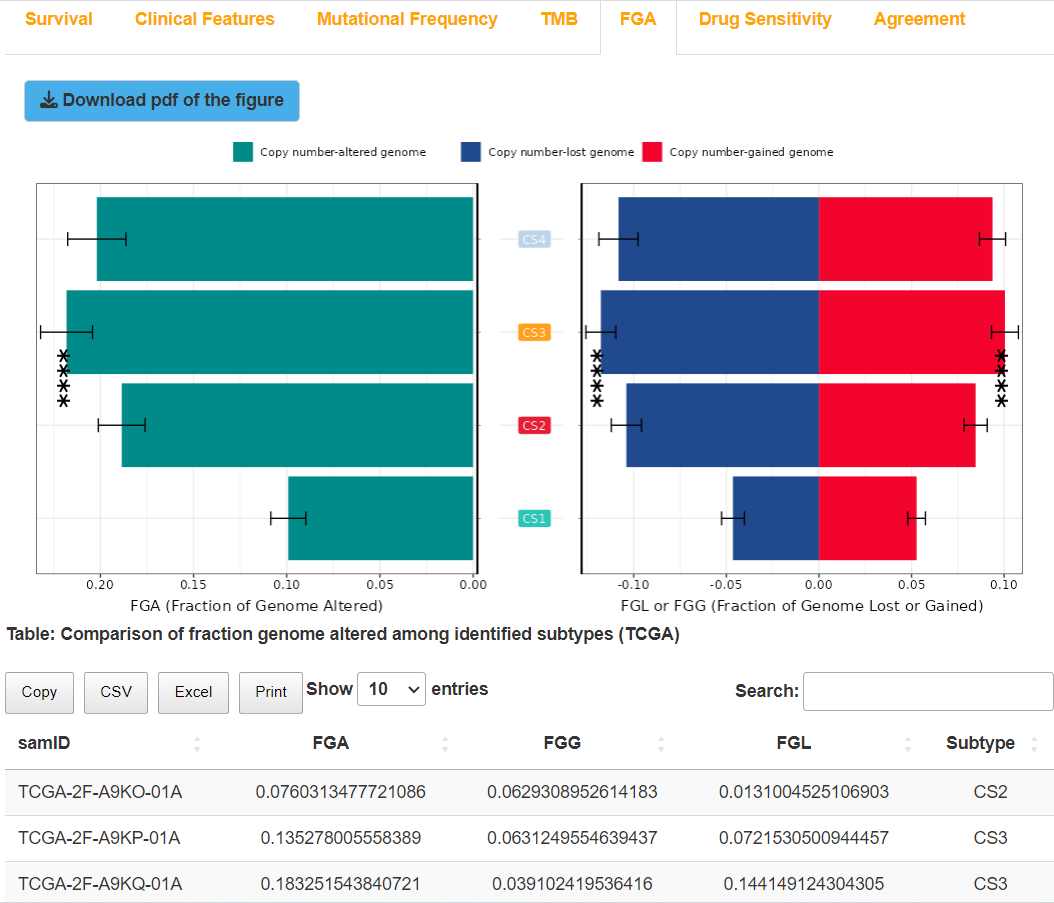


**Figure S19.** Compare the FGA, FGG and FGL among subtypes for TCGA dataset.


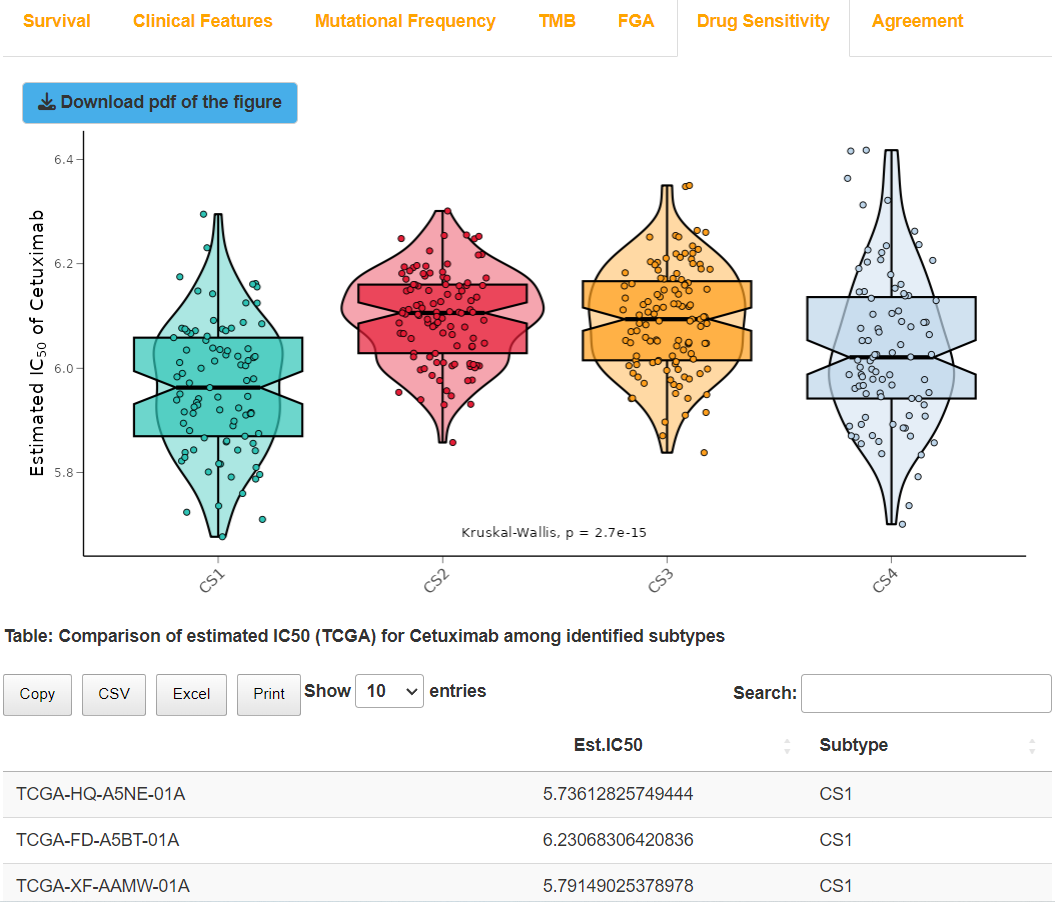


**Figure S20.** Drug sensitivity comparison among subtypes for Cetuximab on TCGA dataset.


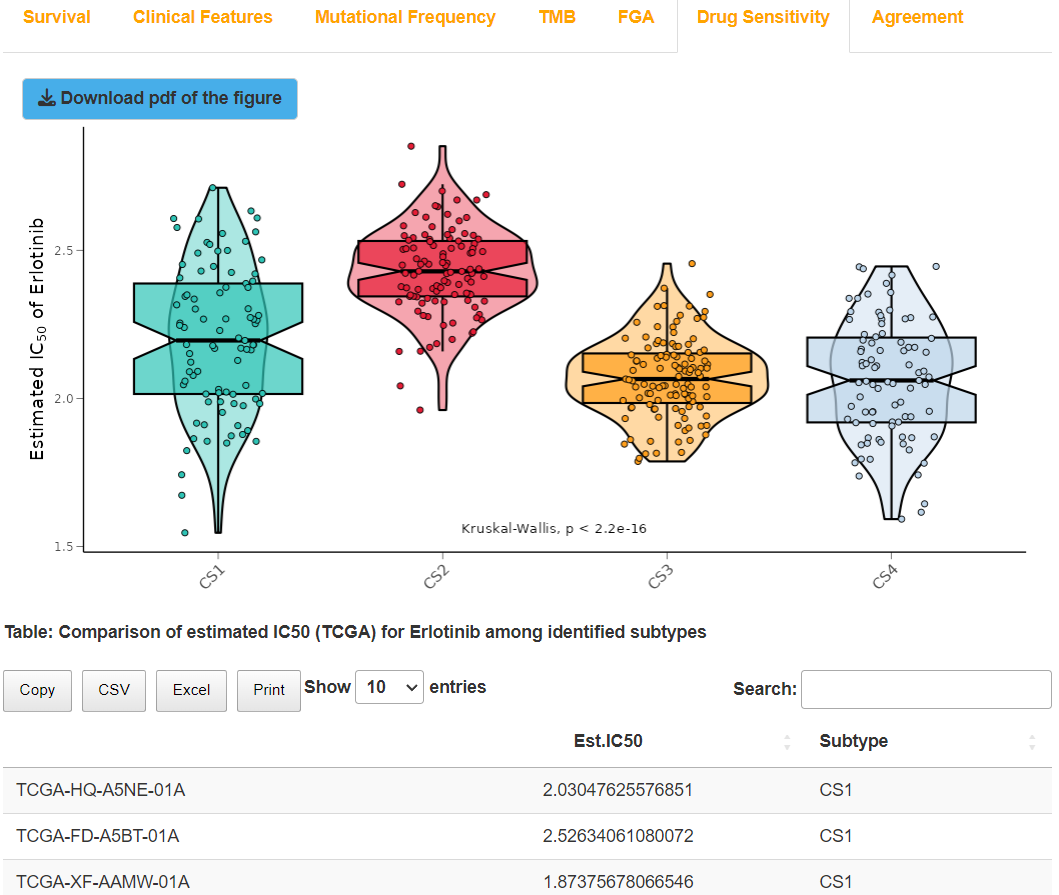


**Figure S21.** Drug sensitivity comparison among subtypes for Erlotinib on TCGA dataset.


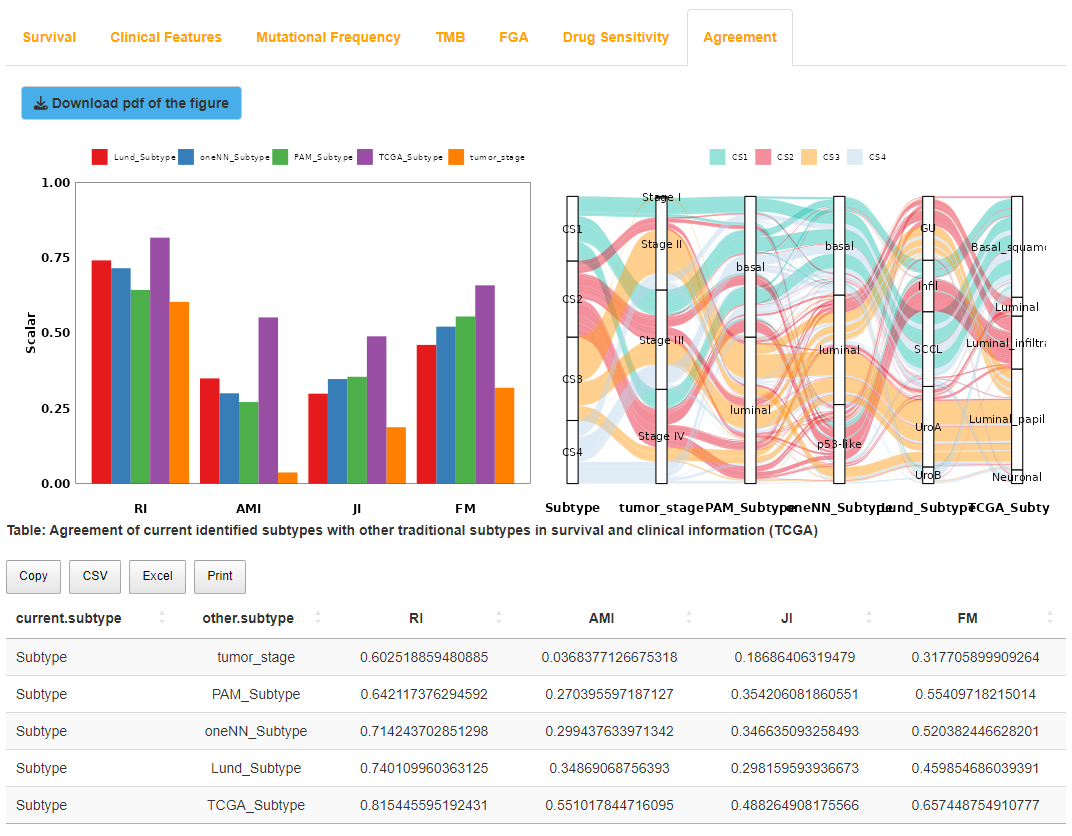


**Figure S22.** Comparison of agreement between obtained subtypes and other traditional subtypes for TCGA dataset. Abbreviations: TCGA, The Cancer Genome Atlas; TMB, total mutation burden; FGA, fraction of genome altered; FGG, fraction of genome gained; FGL, fraction of genome lost. Symbols: ****, 0 < p-value < 0.001; ***, 0.001 ≤ p-value < 0.01; **, 0.01 ≤ p-value < 0.05; *, 0.05 ≤ p-value < 0.1; ., 0.1 ≤ p-value < 1.


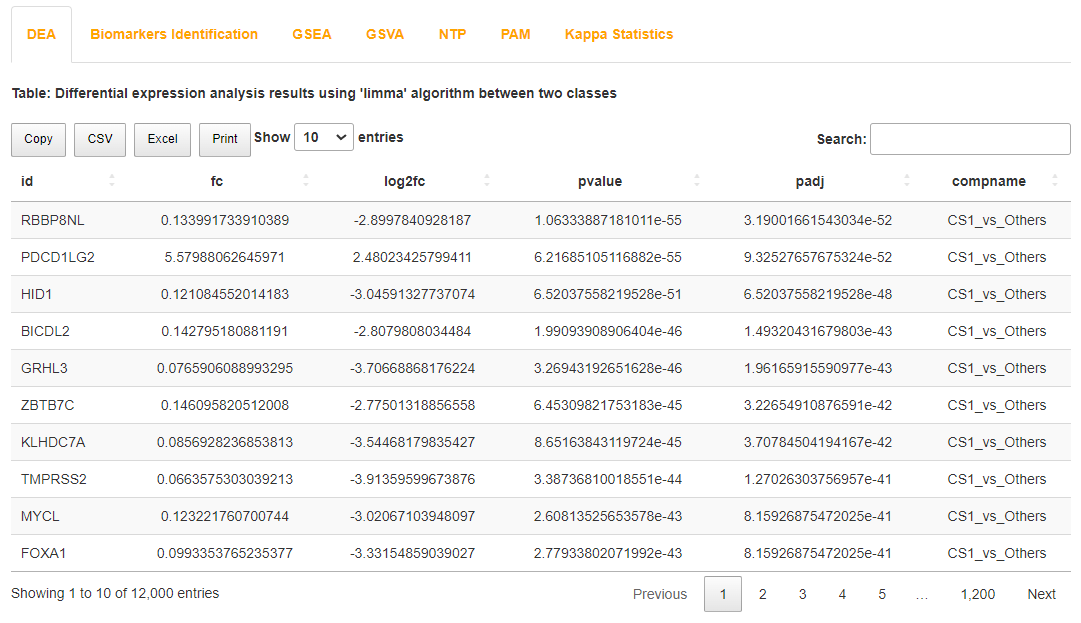


**Figure S23.** The results of differential expression analysis using “limma” algorithm for TCGA dataset.


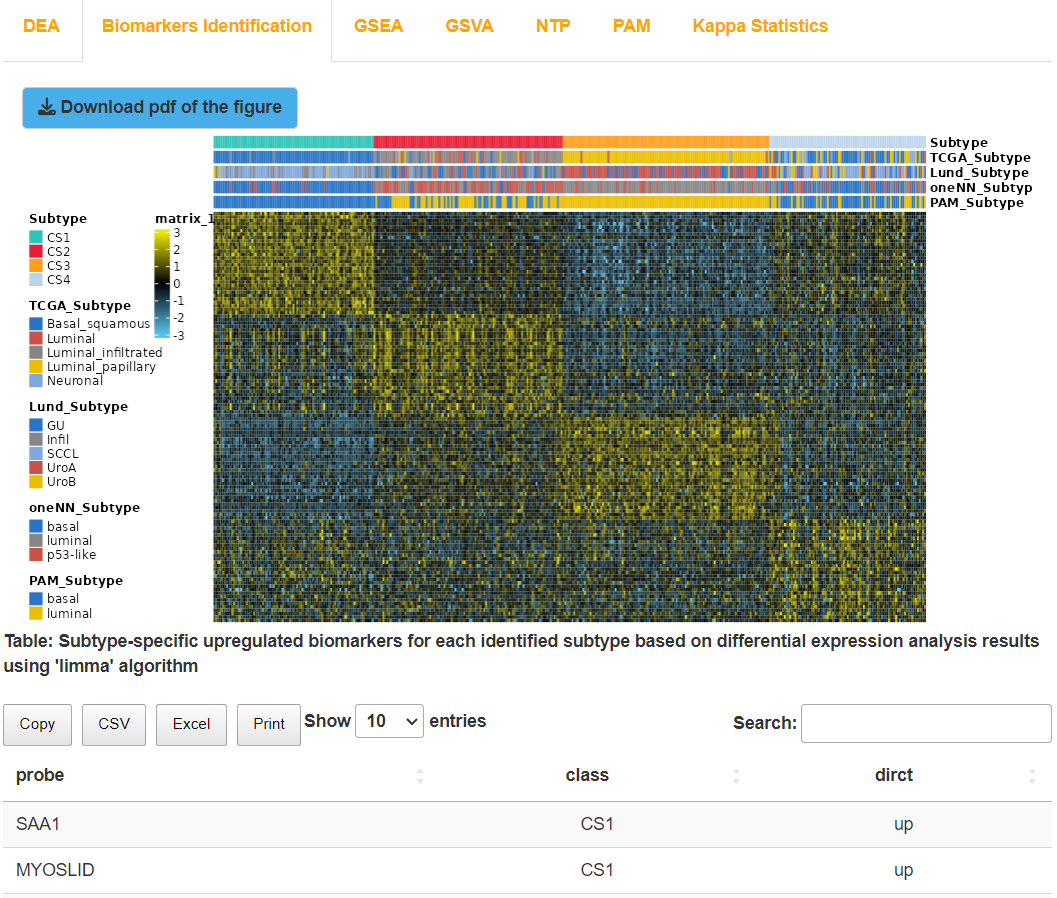


**Figure S24.** Screen up-regulated markers for each subtype on TCGA dataset.


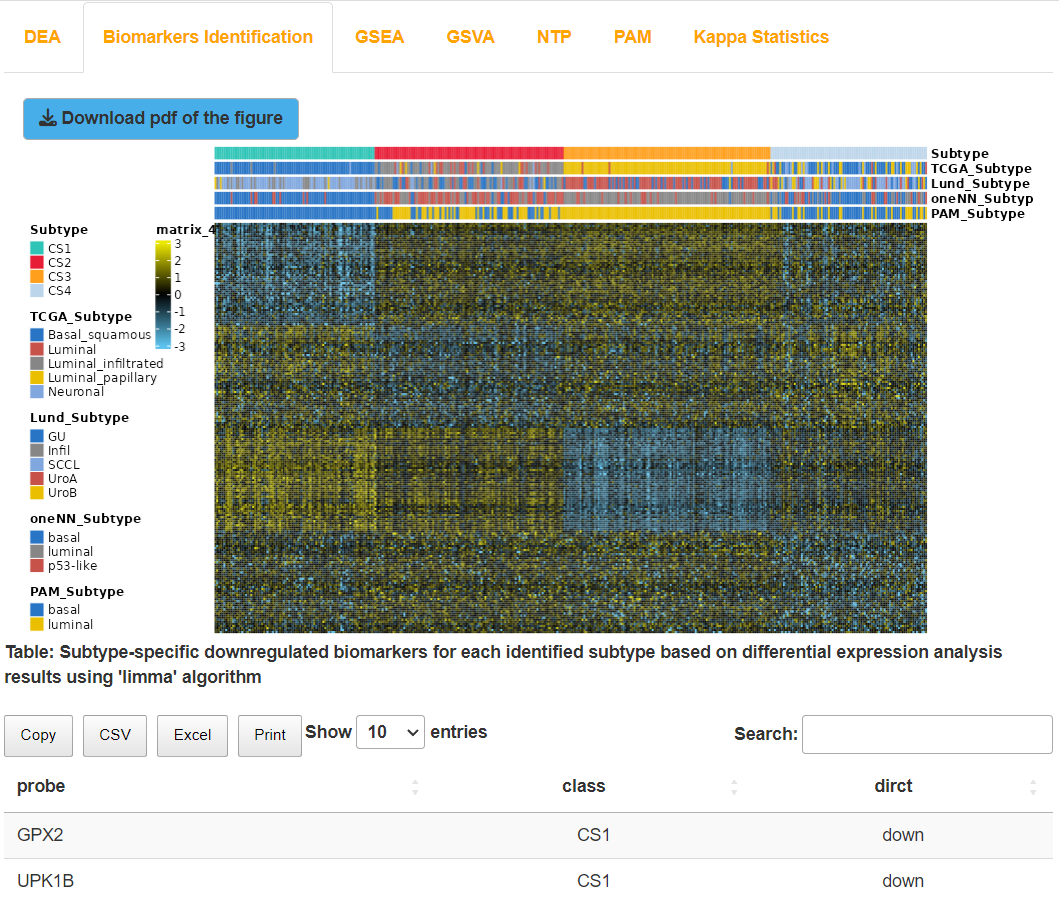


**Figure S25.** Screen down-regulated markers for each subtype on TCGA dataset.


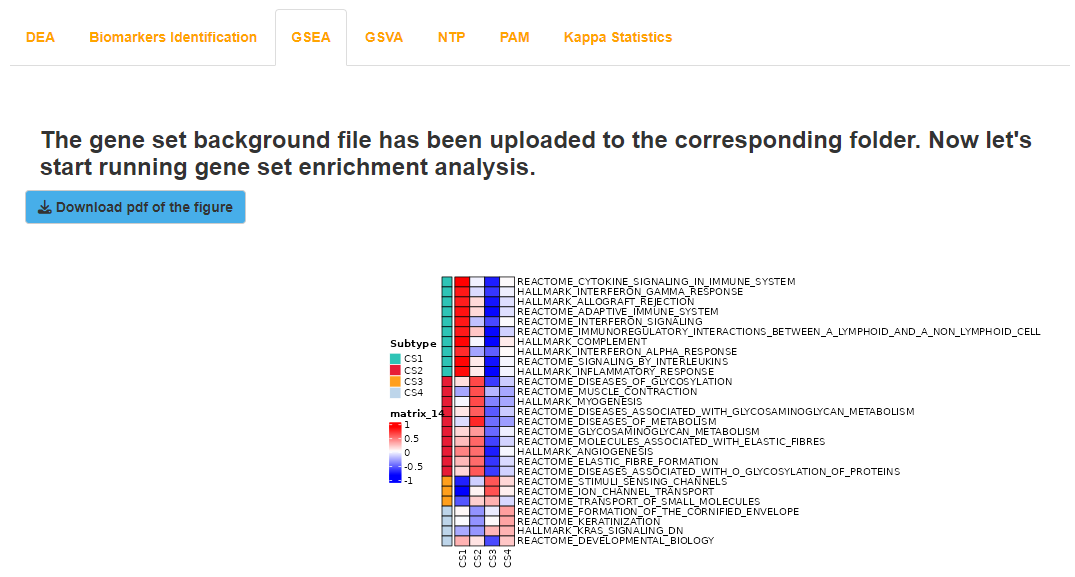


**Figure S26.** A heatmap to show the enrichment scores of the screening up-regulated pathways for each subtype on TCGA dataset.


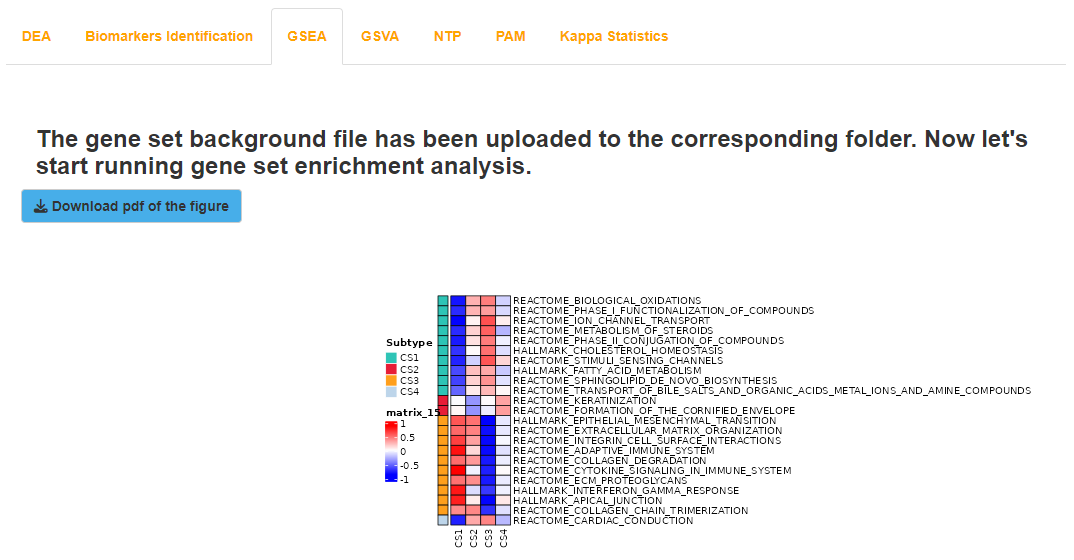


**Figure S27.** A heatmap to show the enrichment scores of the screening down-regulated pathways for each subtype on TCGA dataset.


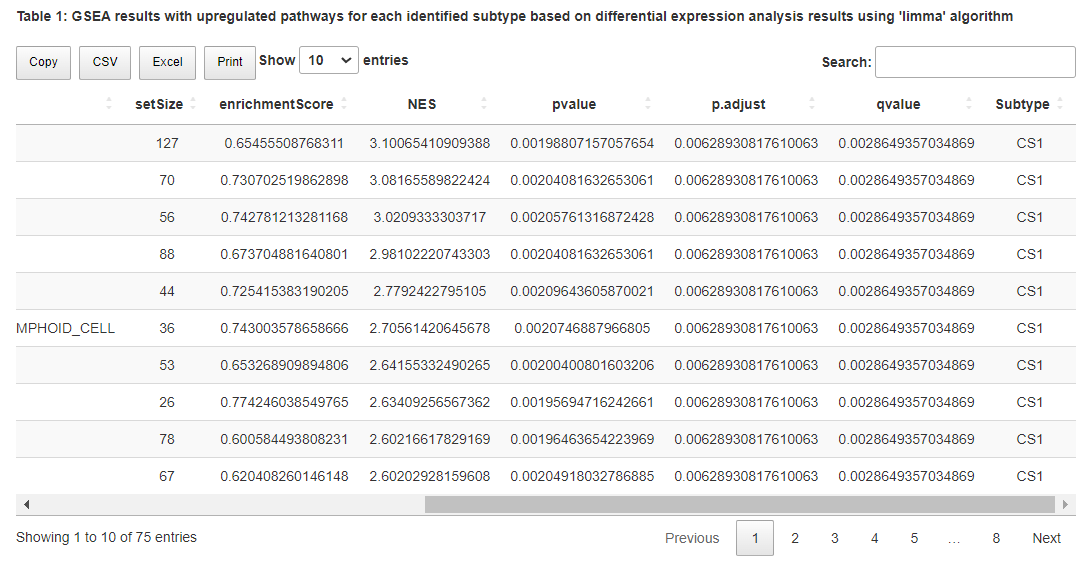


**Figure S28.** Gene set enrichment analysis results for up-regulated pathways on TCGA dataset. Abbreviations: TCGA, The Cancer Genome Atlas.


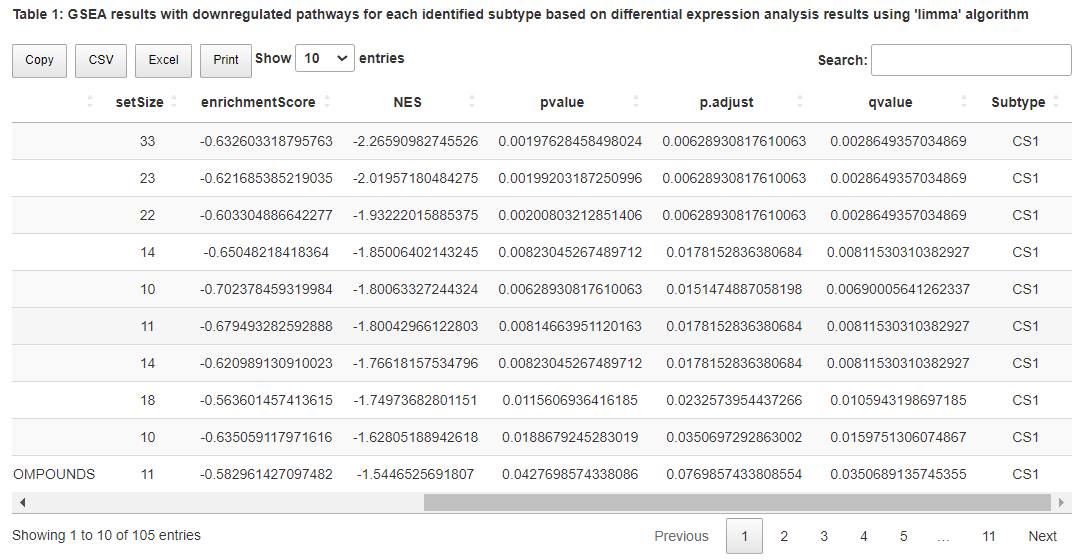


**Figure S29.** Gene set enrichment analysis results for down-regulated pathways on TCGA dataset. Abbreviations: TCGA, The Cancer Genome Atlas.


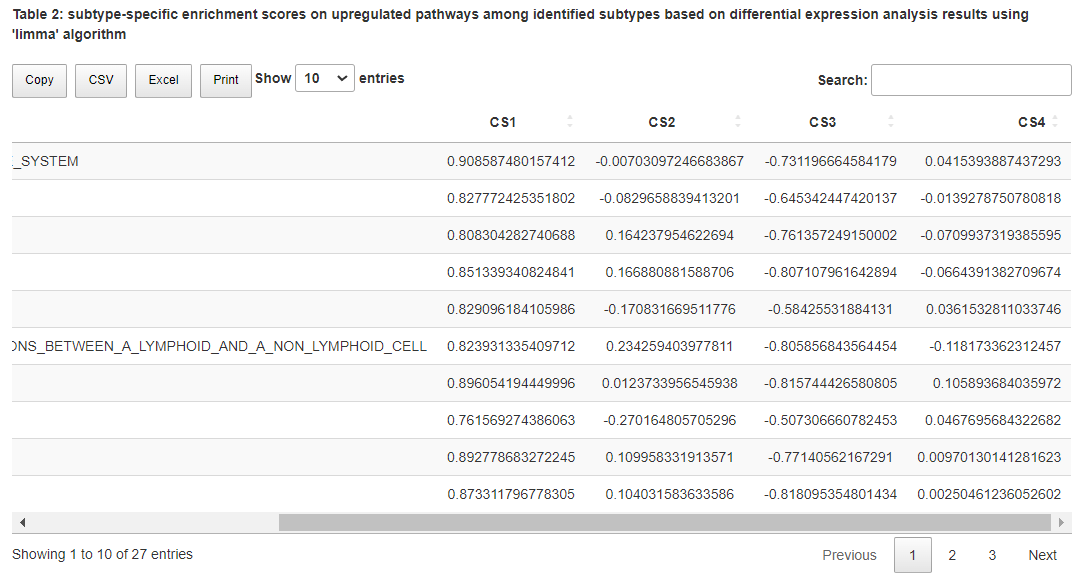


**Figure S30.** Enrichment scores for up-regulated pathways on TCGA dataset. Abbreviations: TCGA, The Cancer Genome Atlas.


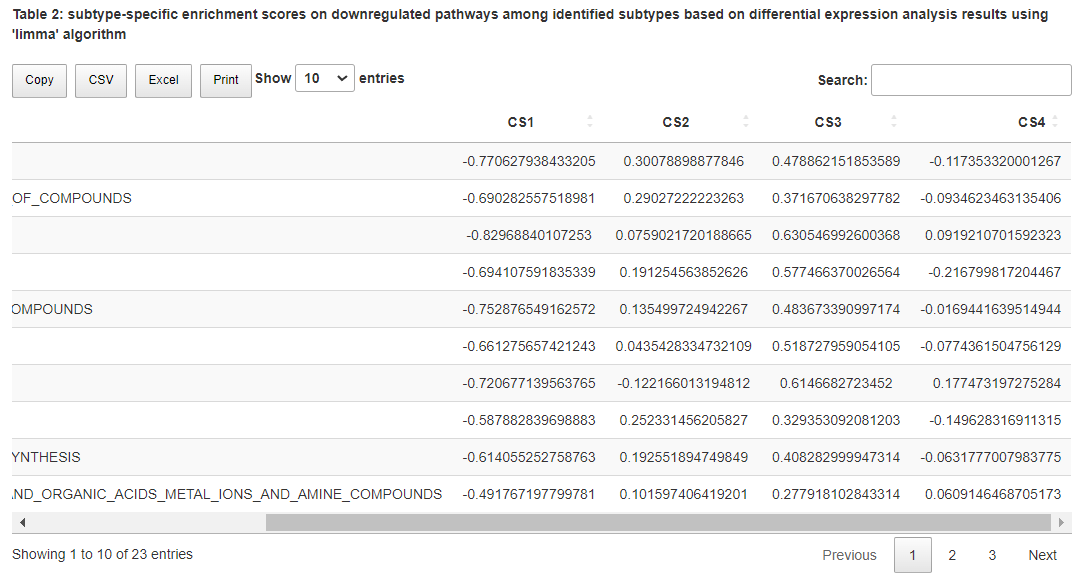


**Figure S31.** Enrichment scores for down-regulated pathways on TCGA dataset. Abbreviations: TCGA, The Cancer Genome Atlas.


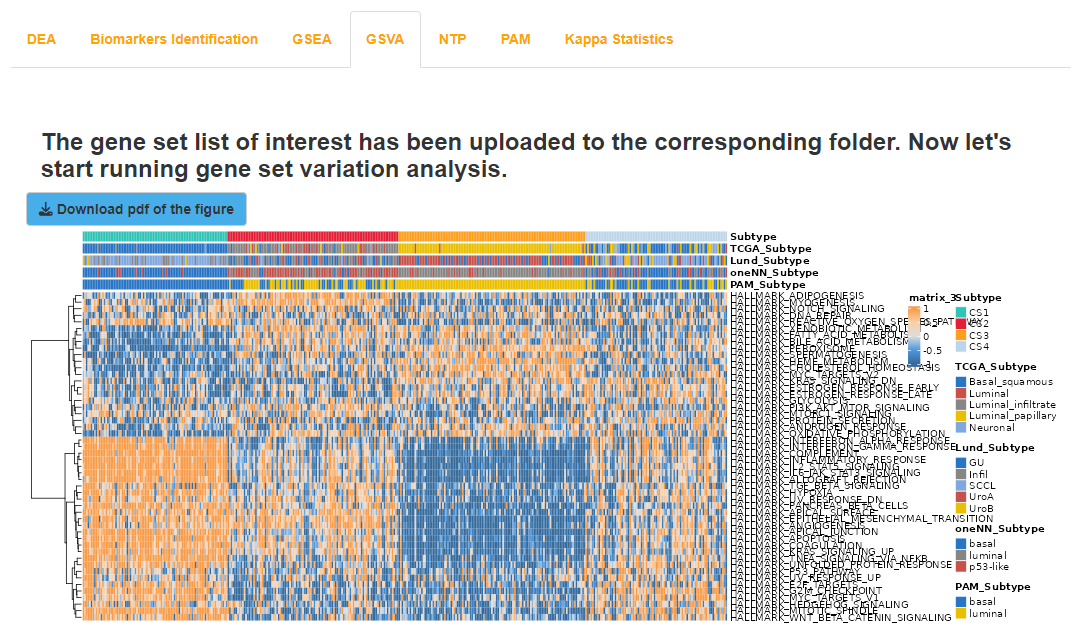


**Figure S32.** A heatmap to show the enrichment scores of the pathways of interest for each sample on TCGA dataset. Abbreviations: TCGA, The Cancer Genome Atlas.


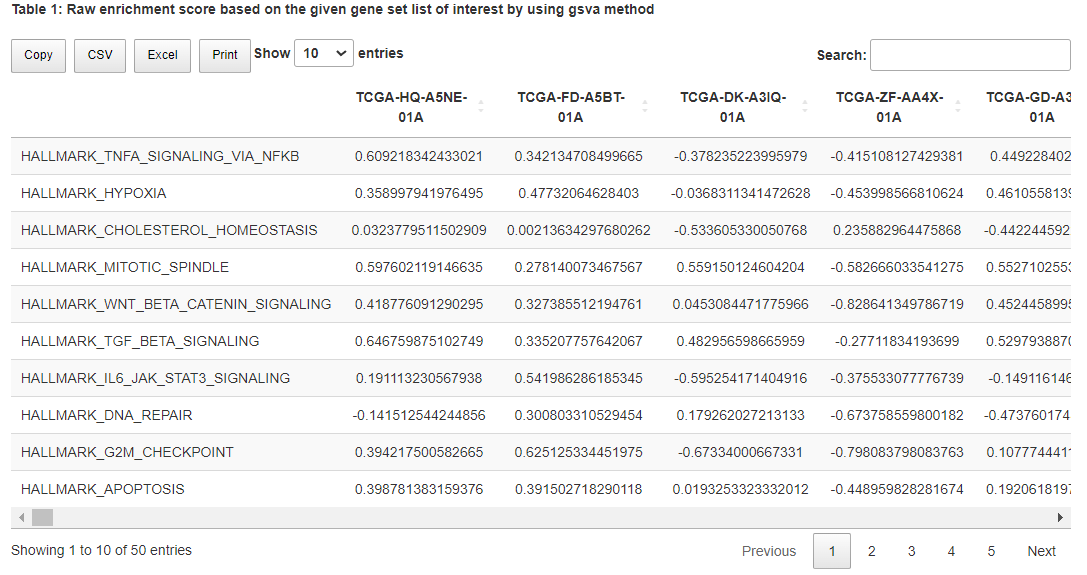


**Figure S33.** Raw enrichment scores for pathways of interest on TCGA dataset. Abbreviations: TCGA, The Cancer Genome Atlas.


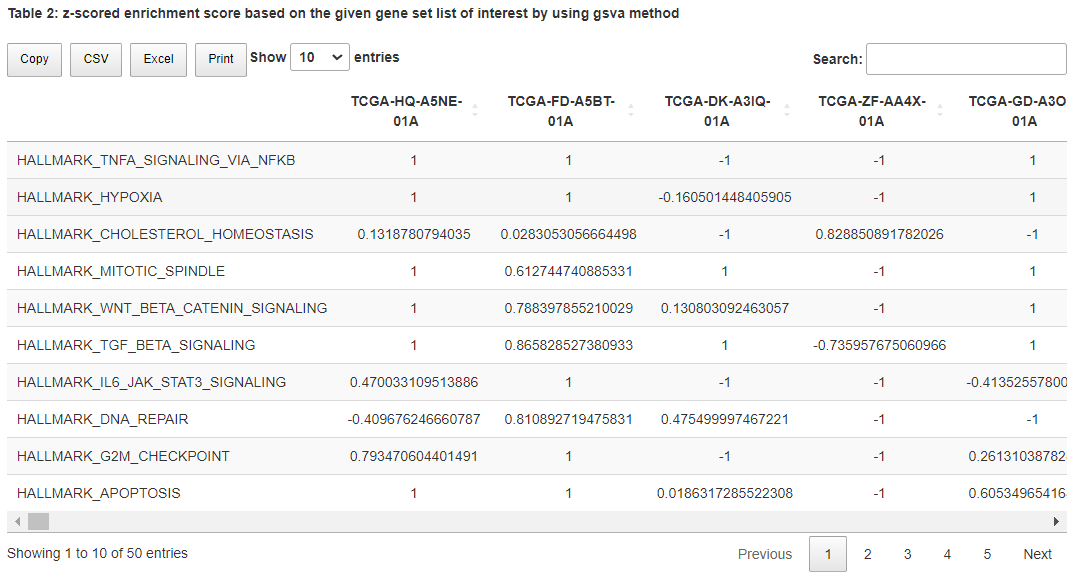


**Figure S34.** z-scored enrichment scores for pathways of interest on TCGA dataset. Abbreviations: TCGA, The Cancer Genome Atlas.


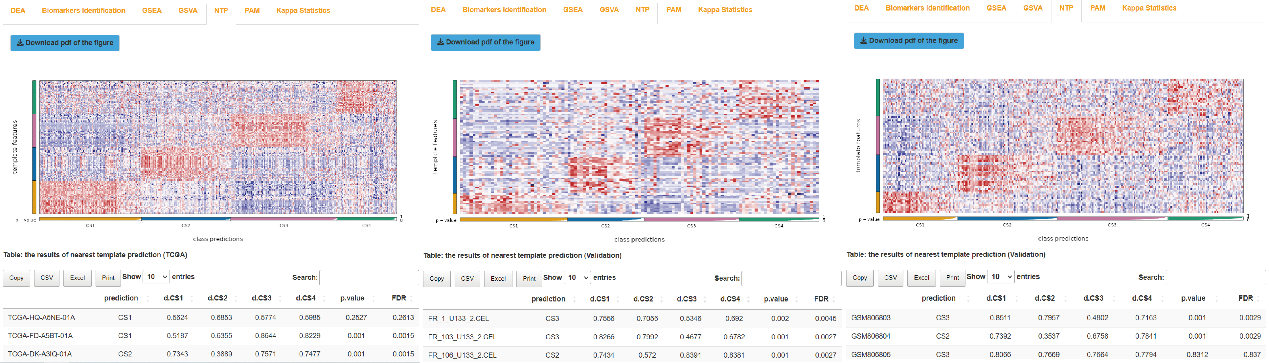


**Figure S35.** Tables to show the subtype prediction results through NTP method as well as heatmaps to evaluate the consistency between prediction results and templates for TCGA dataset, “affy” validation dataset and “illumina” validation dataset respectively.


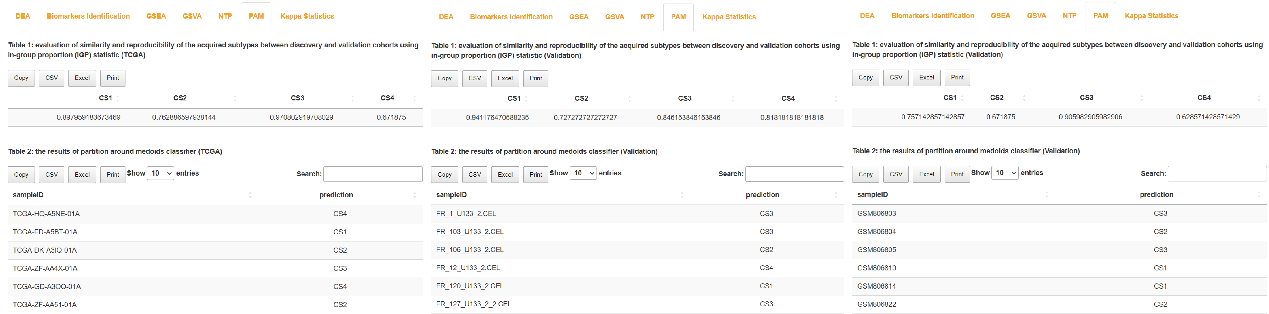


**Figure S36**. Tables to show the subtype prediction results through PAM method as well as tables to display the IGP statistics for each subtype to reflect the similarity and reproducibility between training data and testing data for TCGA dataset, “affy” validation dataset and “illumina” validation dataset separately.


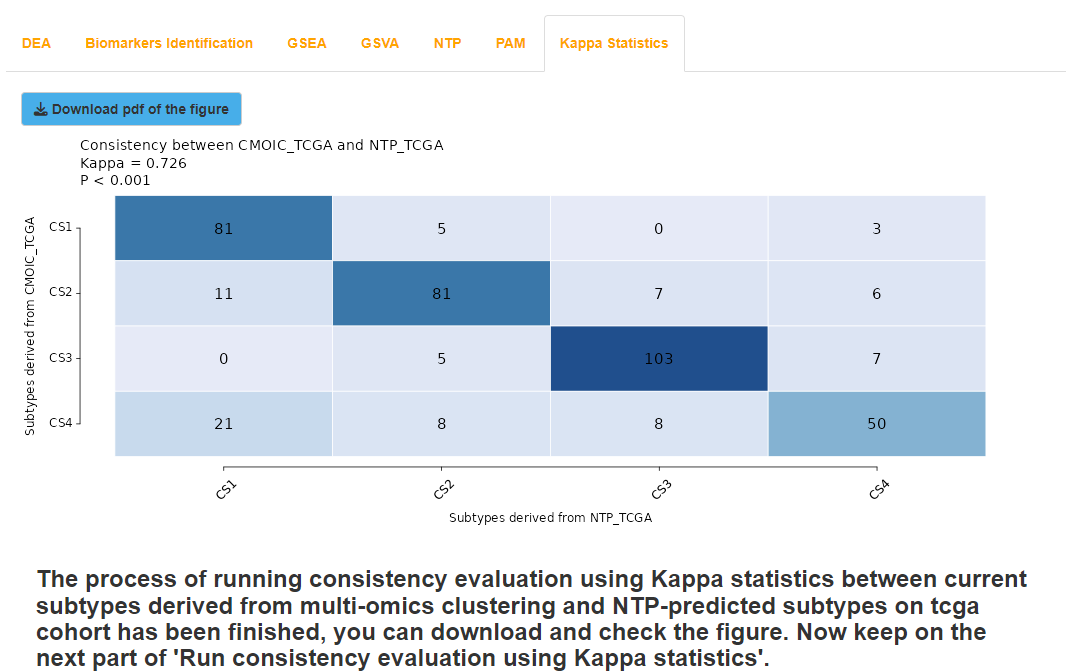


**Figure S37.** A heatmap to evaluate the consistency between clustering results and prediction results derived from NTP method using Kappa statistics for TCGA dataset.


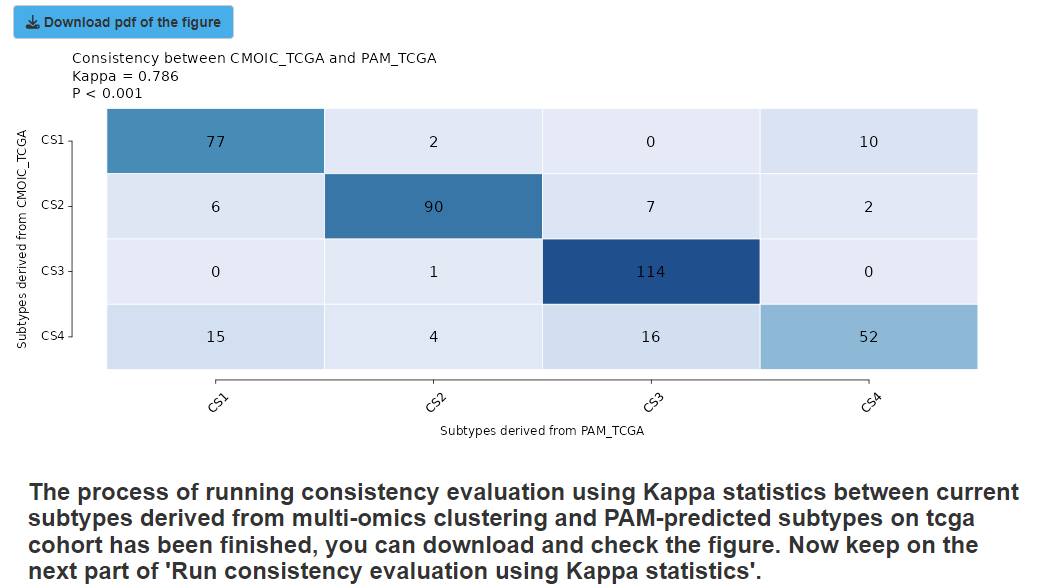


**Figure S38.** A heatmap to evaluate the consistency between clustering results and prediction results derived from PAM method using Kappa statistics for TCGA dataset.


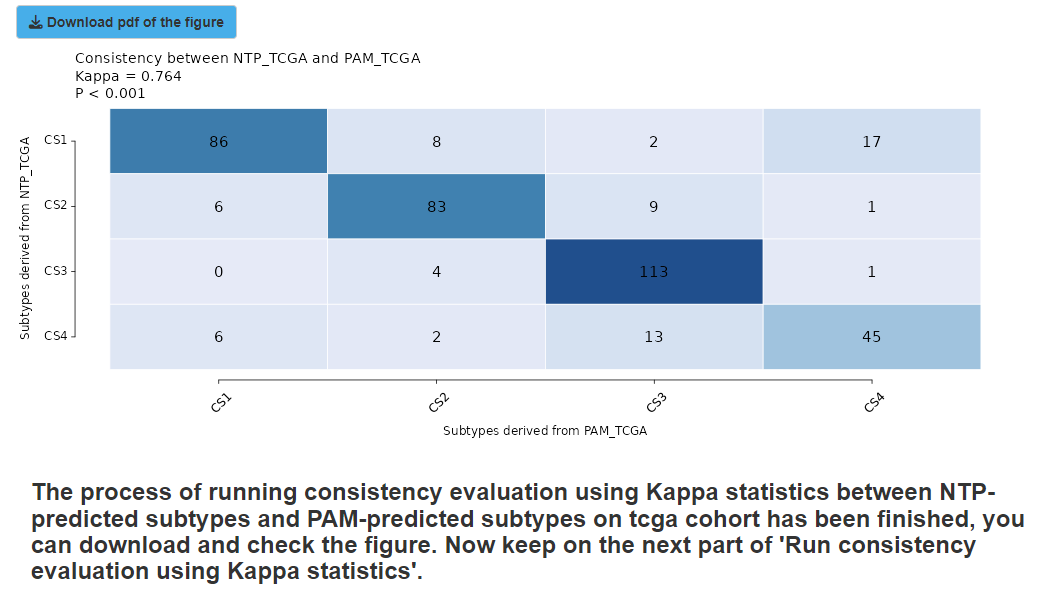


**Figure S39.** A heatmap to evaluate the consistency between prediction results derived from NTP method and PAM method respectively using Kappa statistics for TCGA dataset.


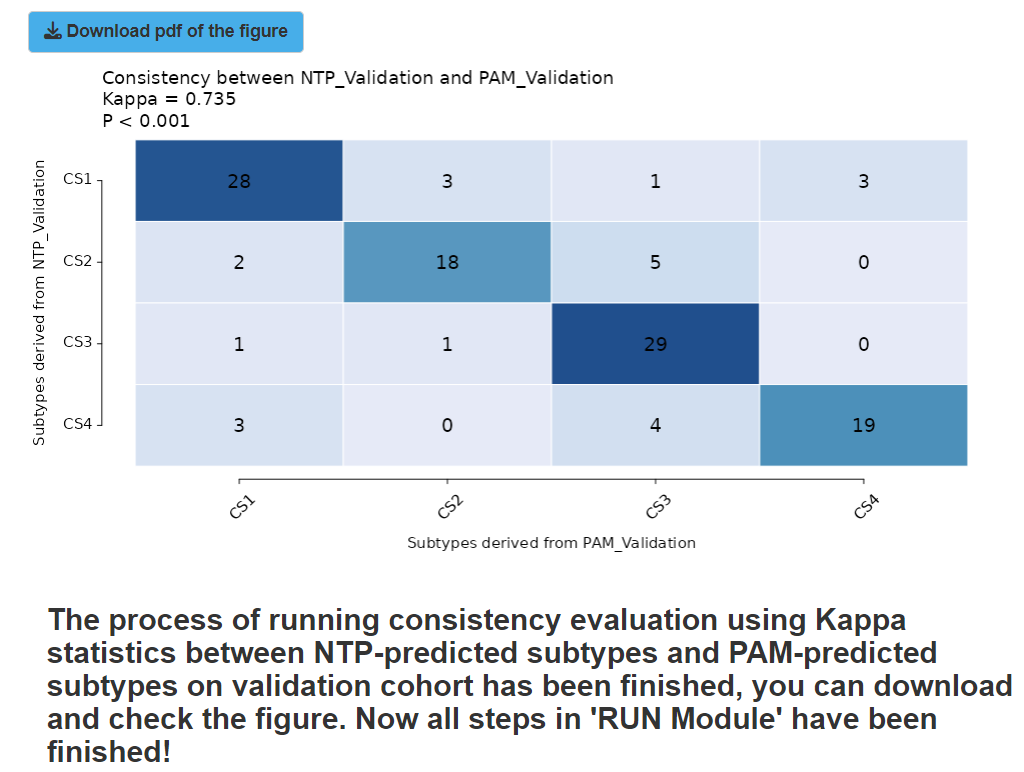


**Figure S40.** A heatmap to evaluate the consistency between prediction results derived from NTP method and PAM method respectively using Kappa statistics for “affy” validation dataset.


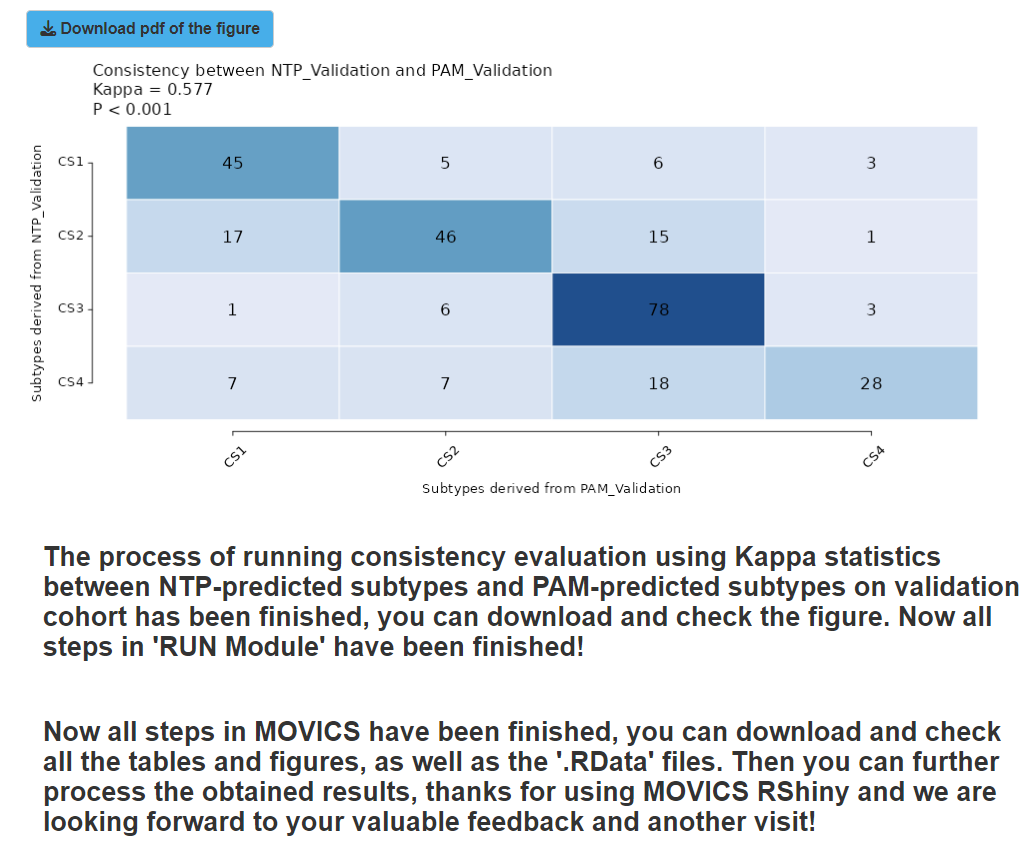


**Figure S41.** A heatmap to evaluate the consistency between prediction results derived from NTP method and PAM method respectively using Kappa statistics for “illumina” validation dataset. Abbreviations: TCGA, The Cancer Genome Atlas; NTP, nearest template prediction; PAM, partition around medoids; IGP, in-group proportion.


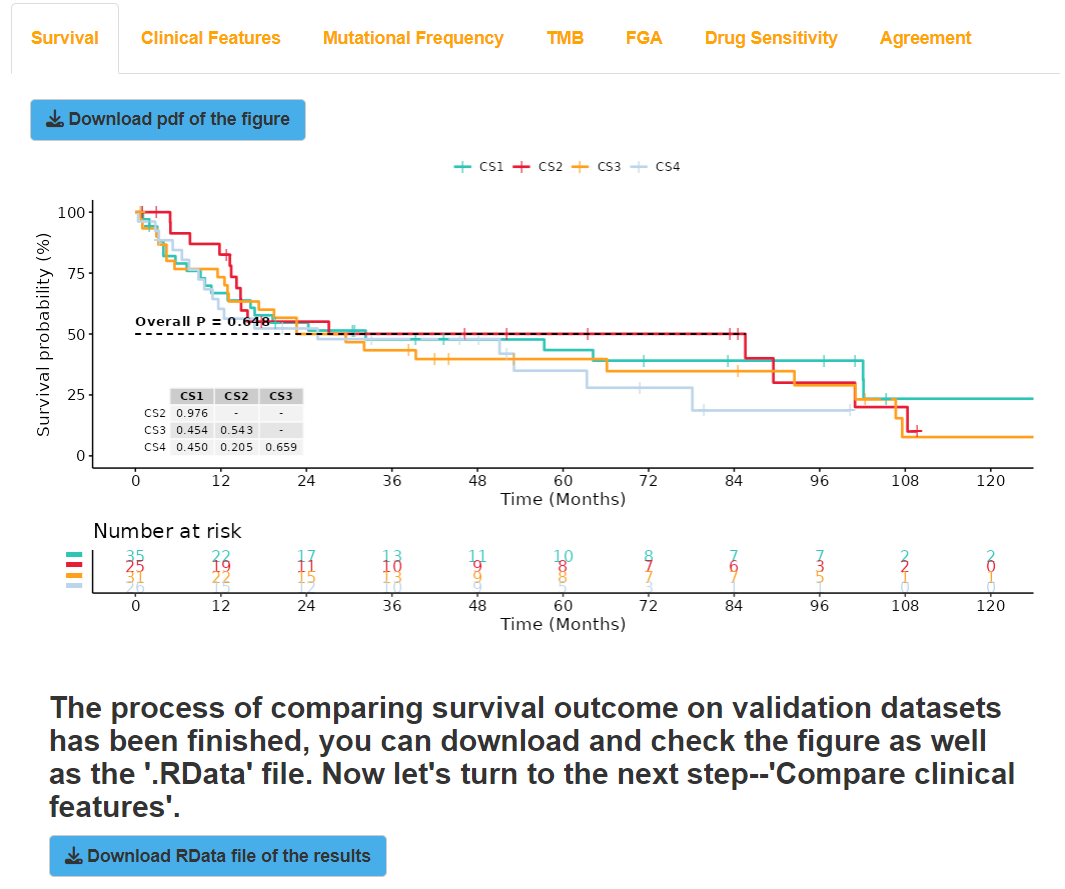


**Figure S42.** Compare the survival differences among subtypes derived from NTP method for “affy” validation dataset. Abbreviations: NTP, nearest template prediction.


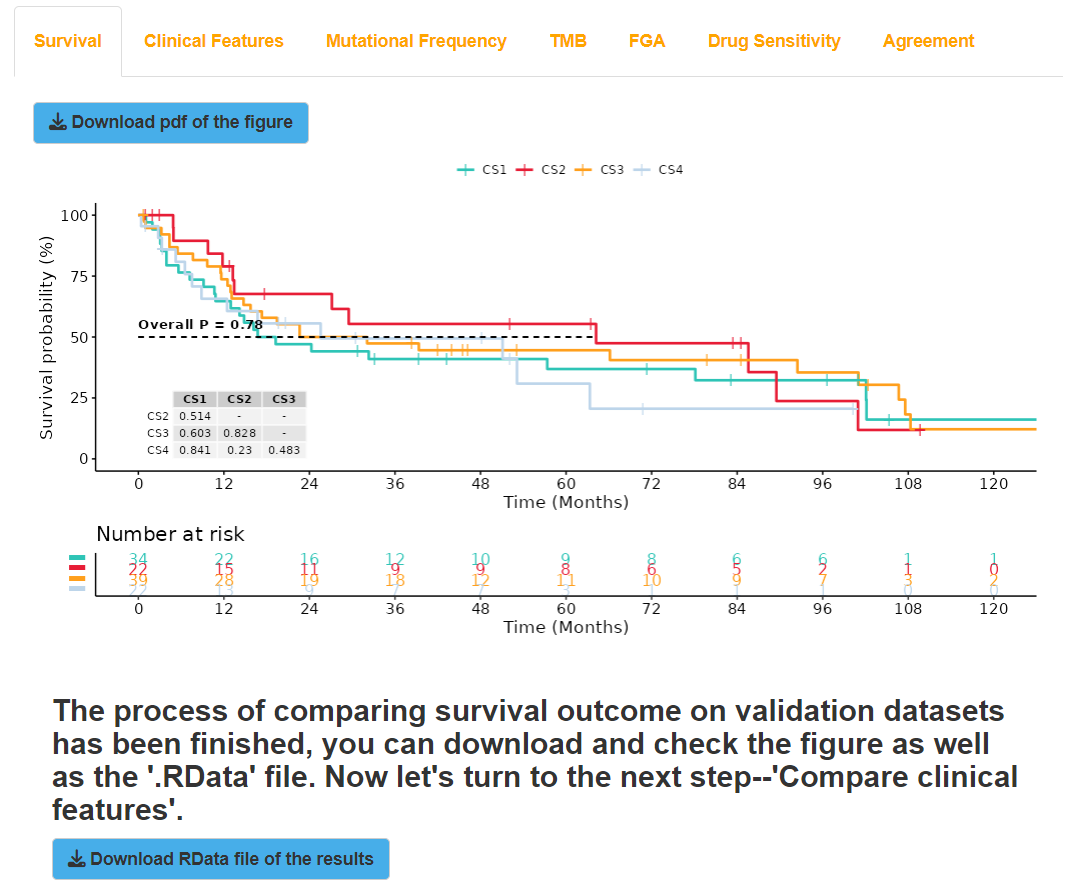


**Figure S43.** Compare the survival differences among subtypes derived from PAM method for “affy” validation dataset. Abbreviations: PAM, partition around medoids.


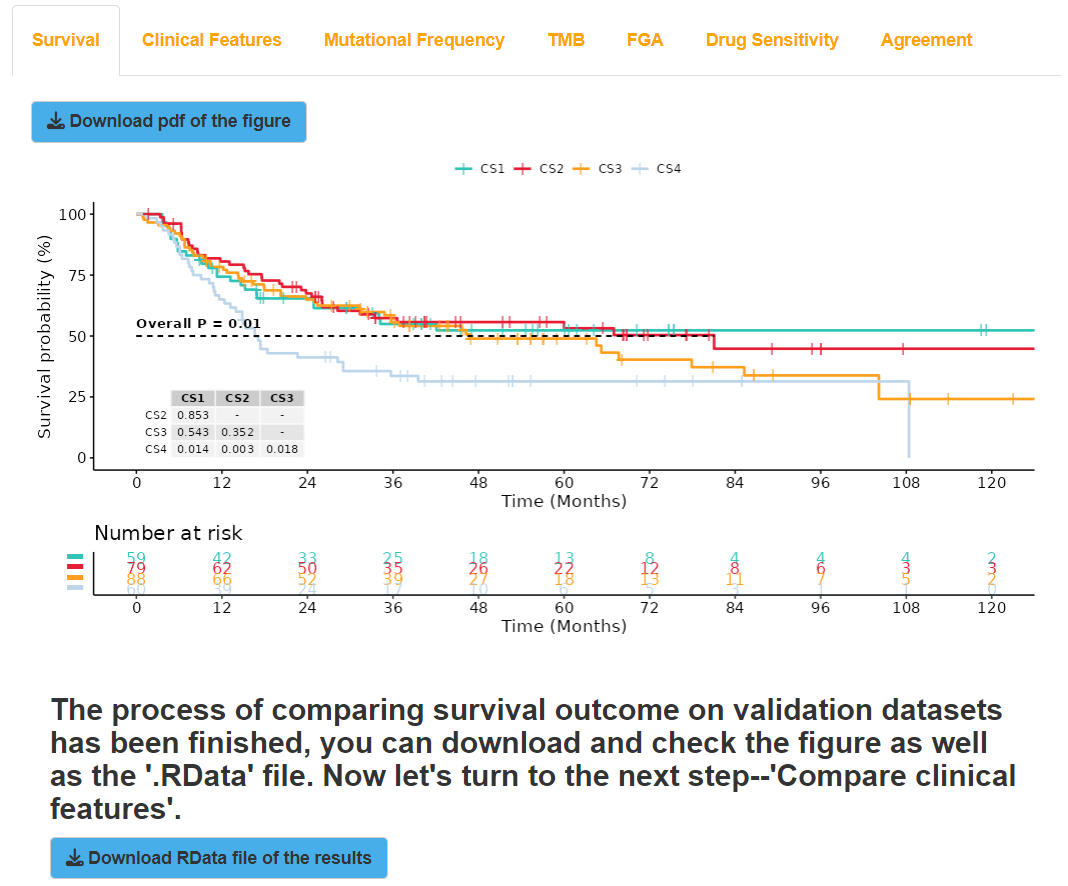


**Figure S44.** Compare the survival differences among subtypes derived from NTP method for “illumina” validation dataset. Abbreviations: NTP, nearest template prediction.


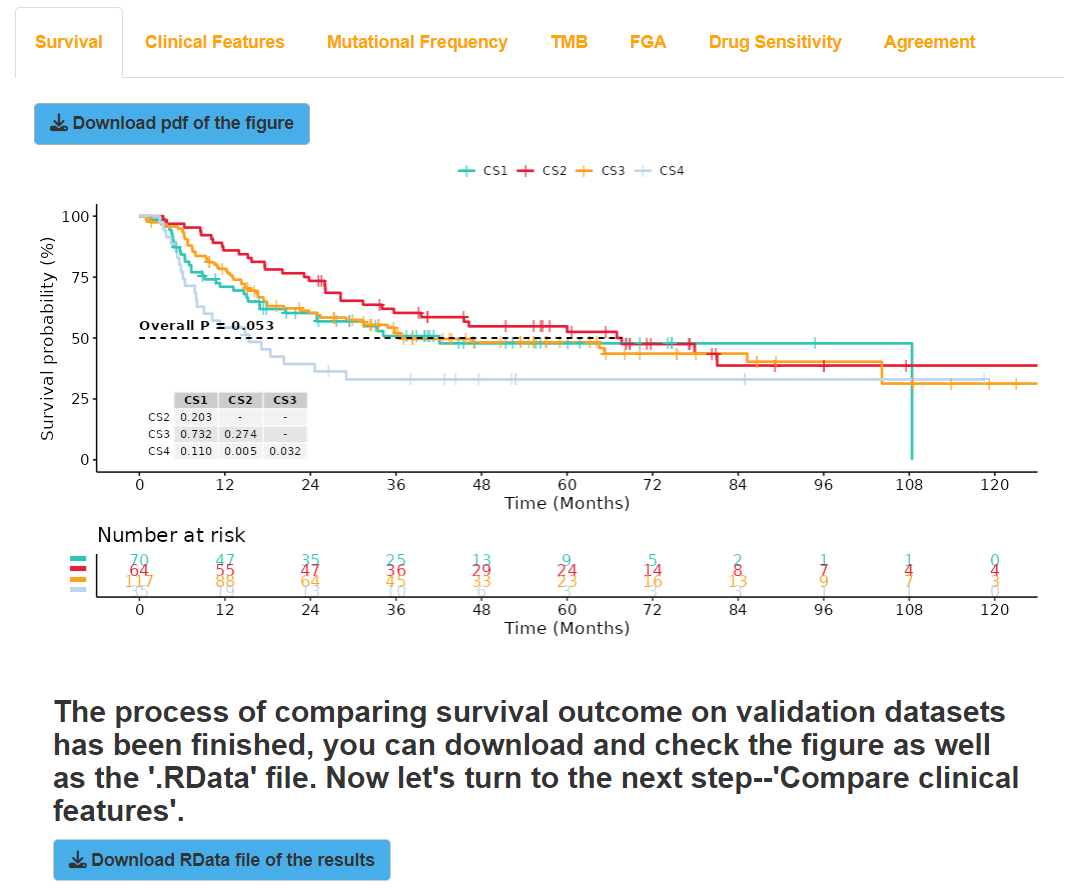


**Figure S45.** Compare the survival differences among subtypes derived from PAM method for “illumina” validation dataset. Abbreviations: PAM, partition around medoids.


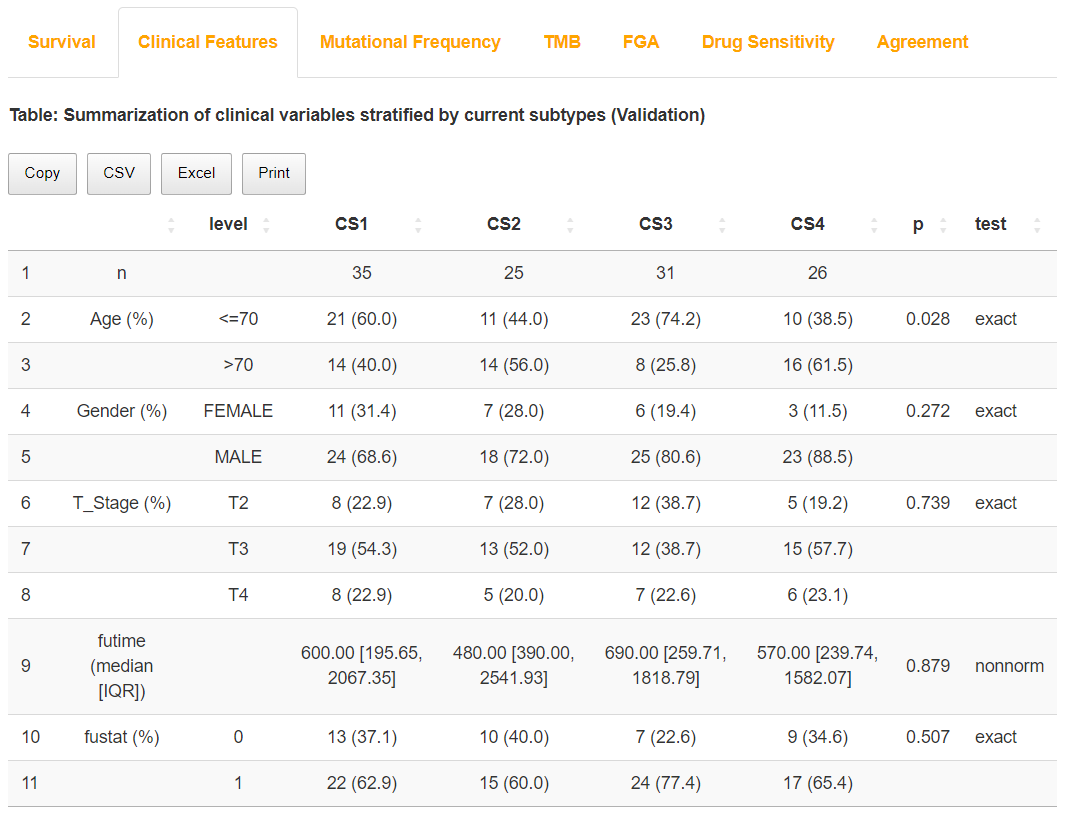


**Figure S46.** Compare the clinical features among subtypes derived from NTP method to find out the clinical features which are significantly correlated with subtypes for “affy” validation dataset. Abbreviations: NTP, nearest template prediction.


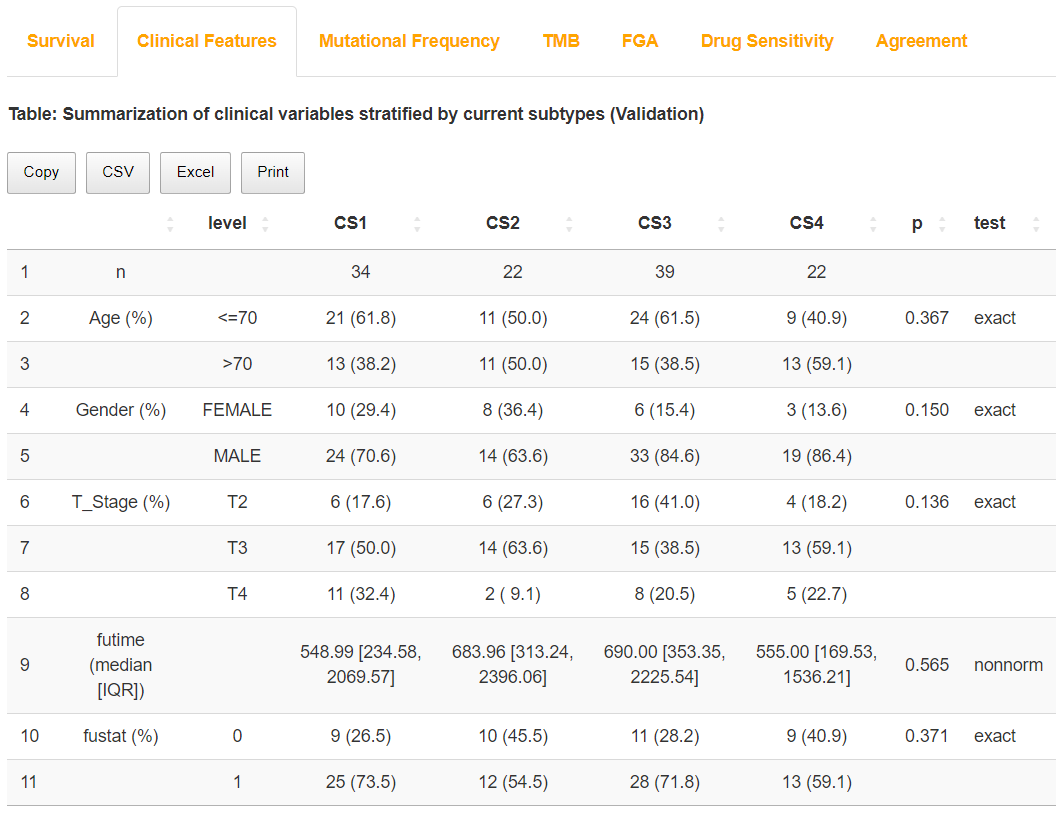


**Figure S47.** Compare the clinical features among subtypes derived from PAM method to find out the clinical features which are significantly correlated with subtypes for “affy” validation dataset. Abbreviations: PAM, partition around medoids.


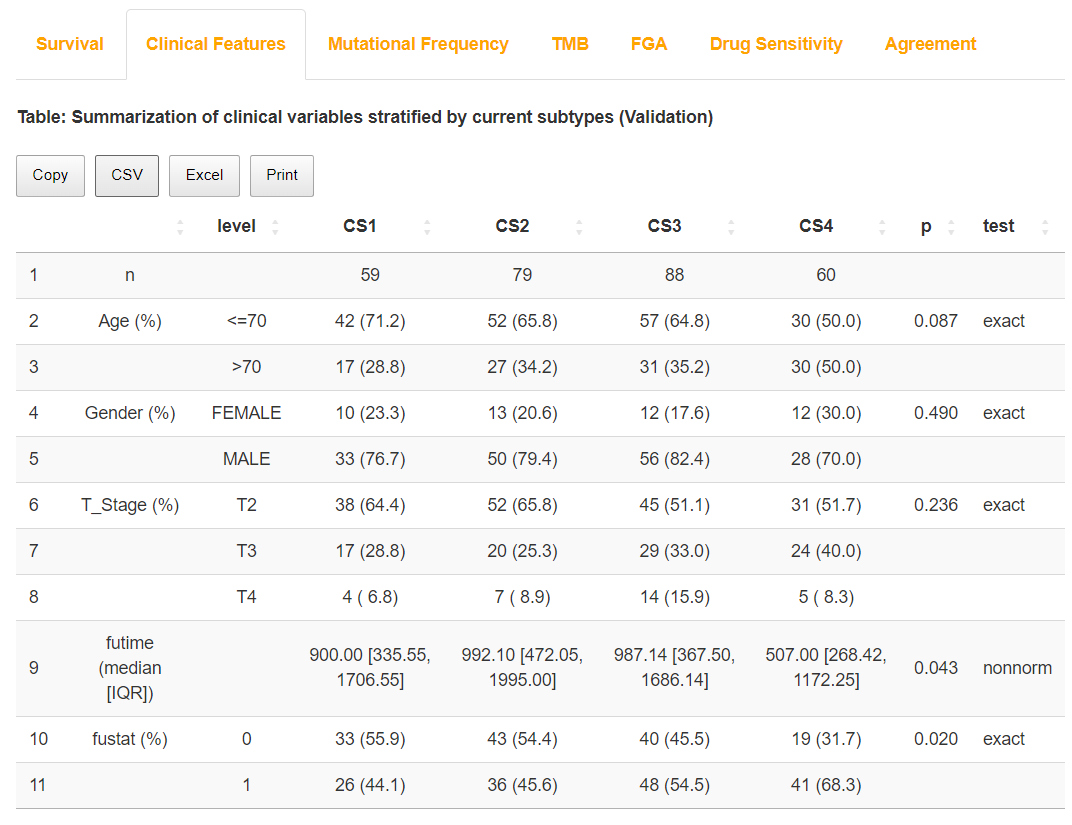


**Figure S48.** Compare the clinical features among subtypes derived from NTP method to find out the clinical features which are significantly correlated with subtypes for “illumina” validation dataset. Abbreviations: NTP, nearest template prediction.


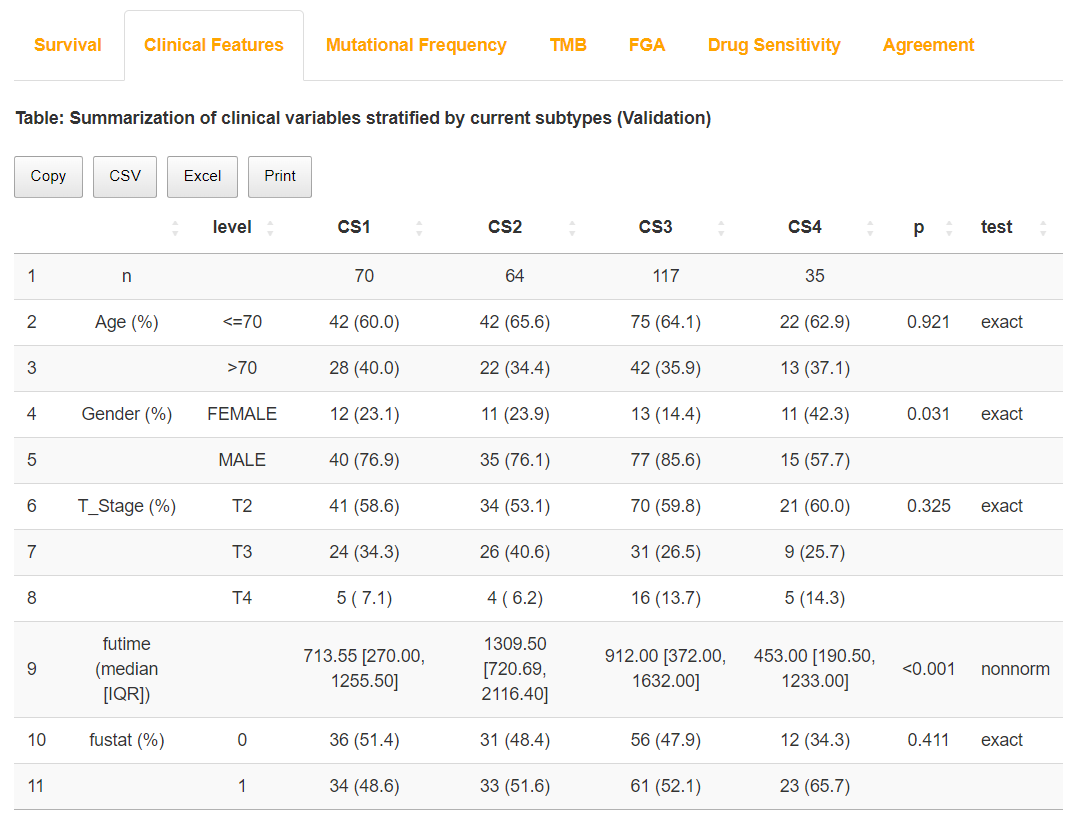


**Figure S49.** Compare the clinical features among subtypes derived from PAM method to find out the clinical features which are significantly correlated with subtypes for “illumina” validation dataset. Abbreviations: PAM, partition around medoids.


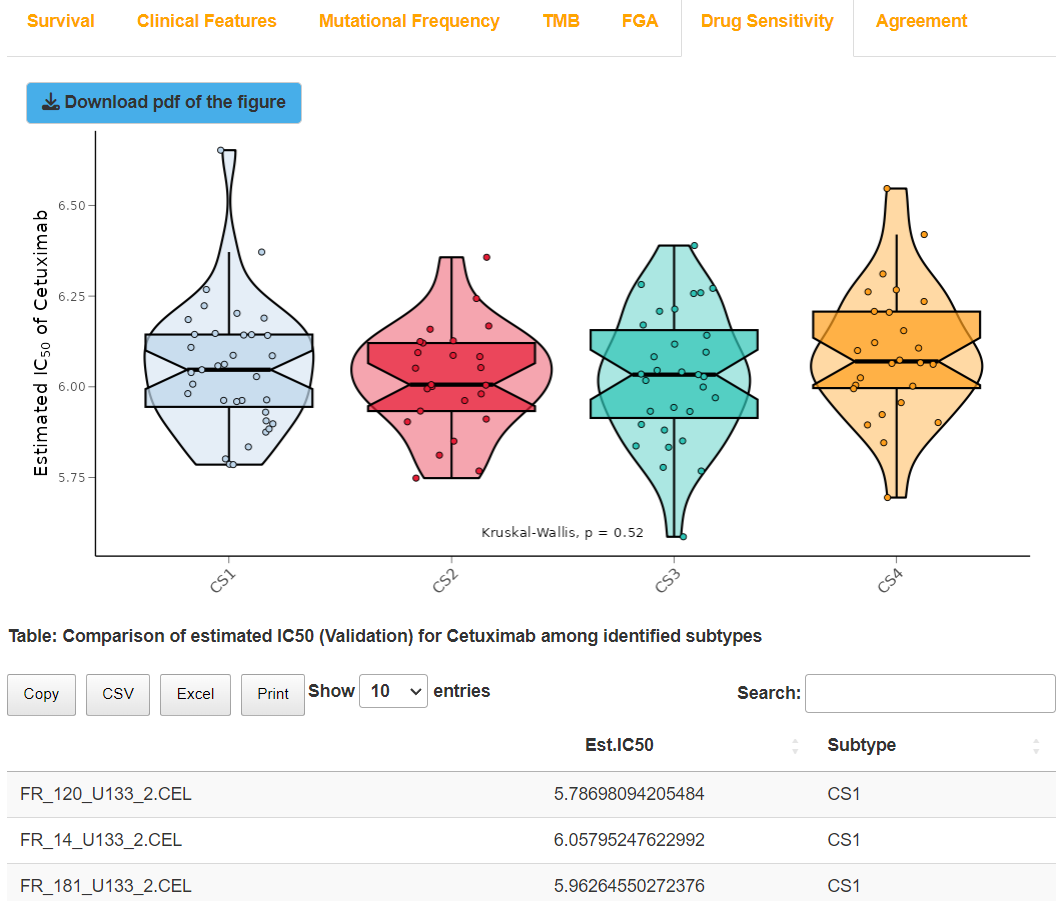


**Figure S50.** Drug sensitivity comparison among subtypes derived from NTP method for Cetuximab on “affy” validation dataset. Abbreviations: NTP, nearest template prediction.


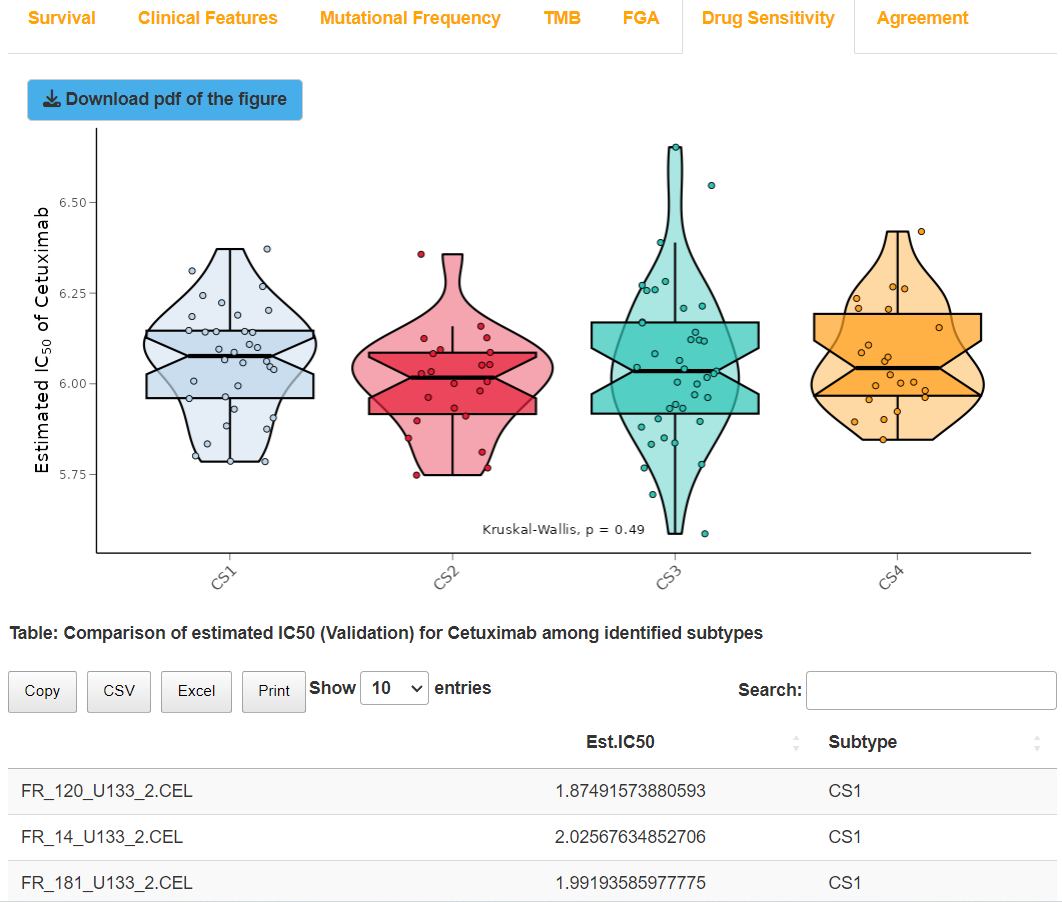


**Figure S51.** Drug sensitivity comparison among subtypes derived from PAM method for Cetuximab on “affy” validation dataset. Abbreviations: PAM, partition around medoids.


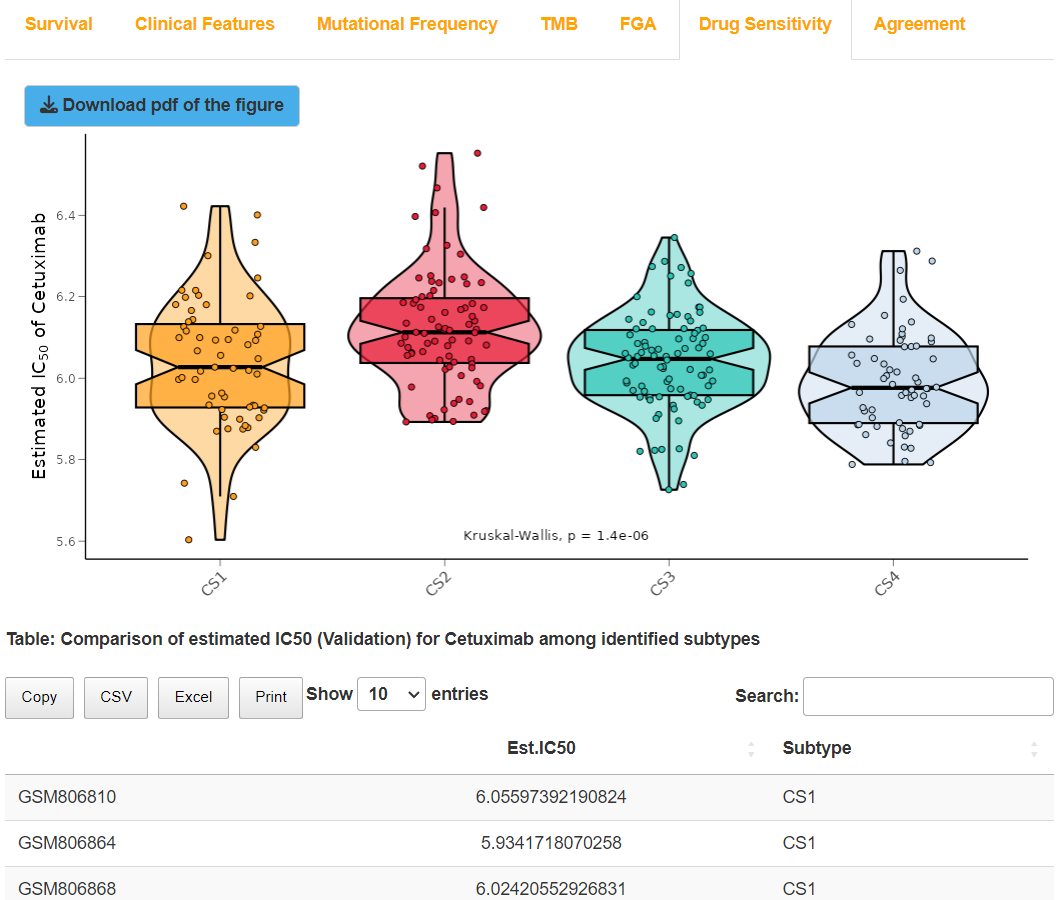


**Figure S52.** Drug sensitivity comparison among subtypes derived from NTP method for Cetuximab on “illumina” validation dataset. Abbreviations: NTP, nearest template prediction.


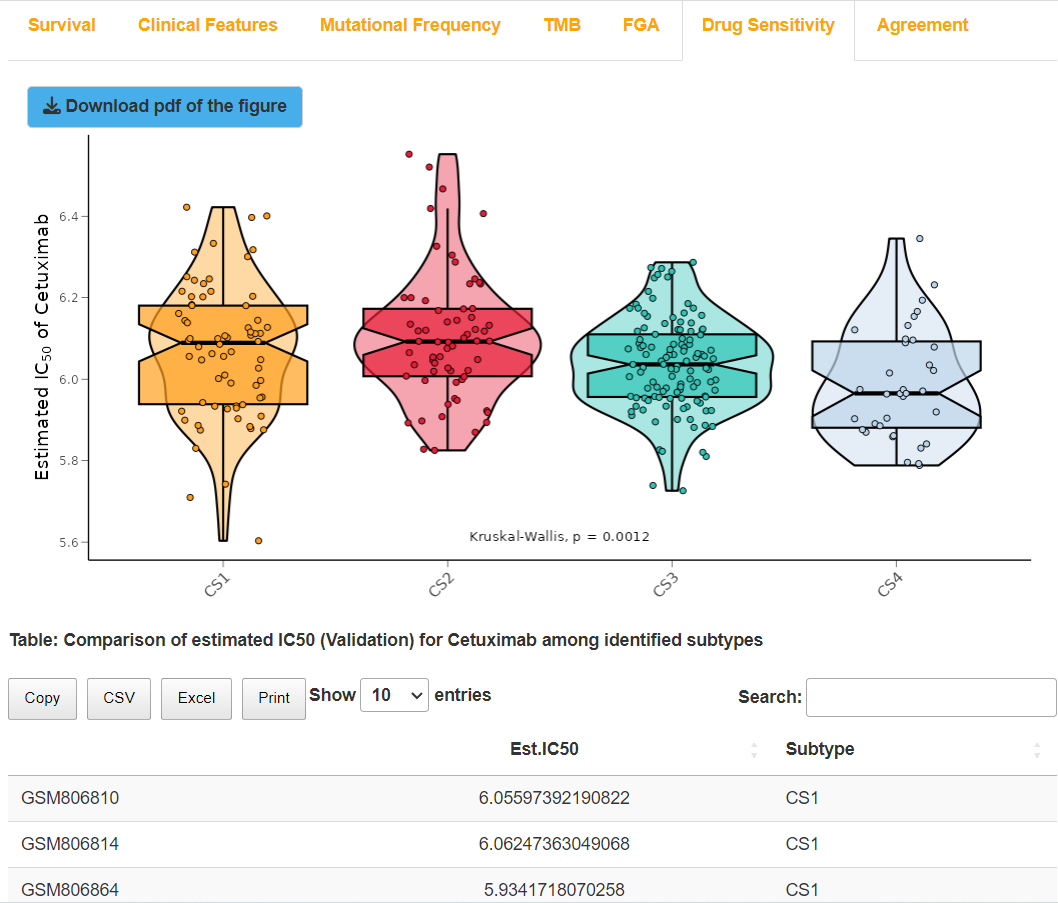


**Figure S53.** Drug sensitivity comparison among subtypes derived from PAM method for Cetuximab on “illumina” validation dataset. Abbreviations: PAM, partition around medoids.


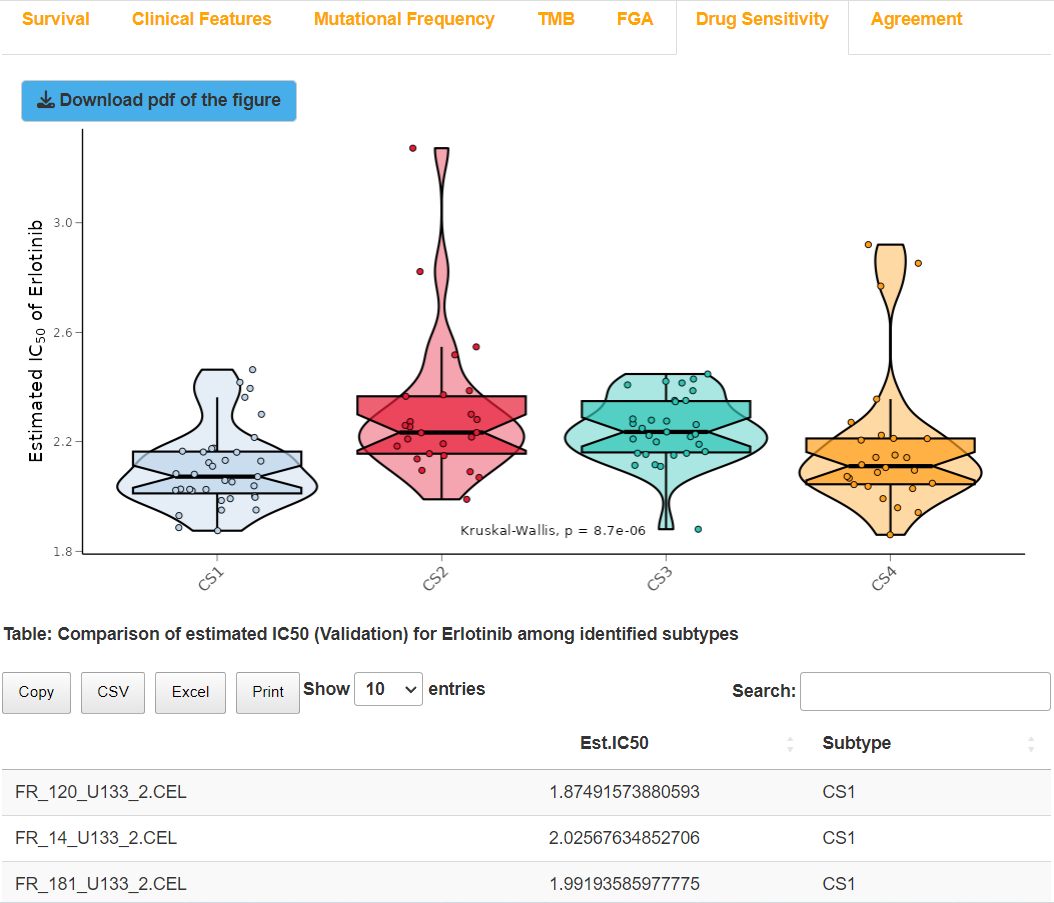


**Figure S54.** Drug sensitivity comparison among subtypes derived from NTP method for Erlotinib on “affy” validation dataset. Abbreviations: NTP, nearest template prediction.


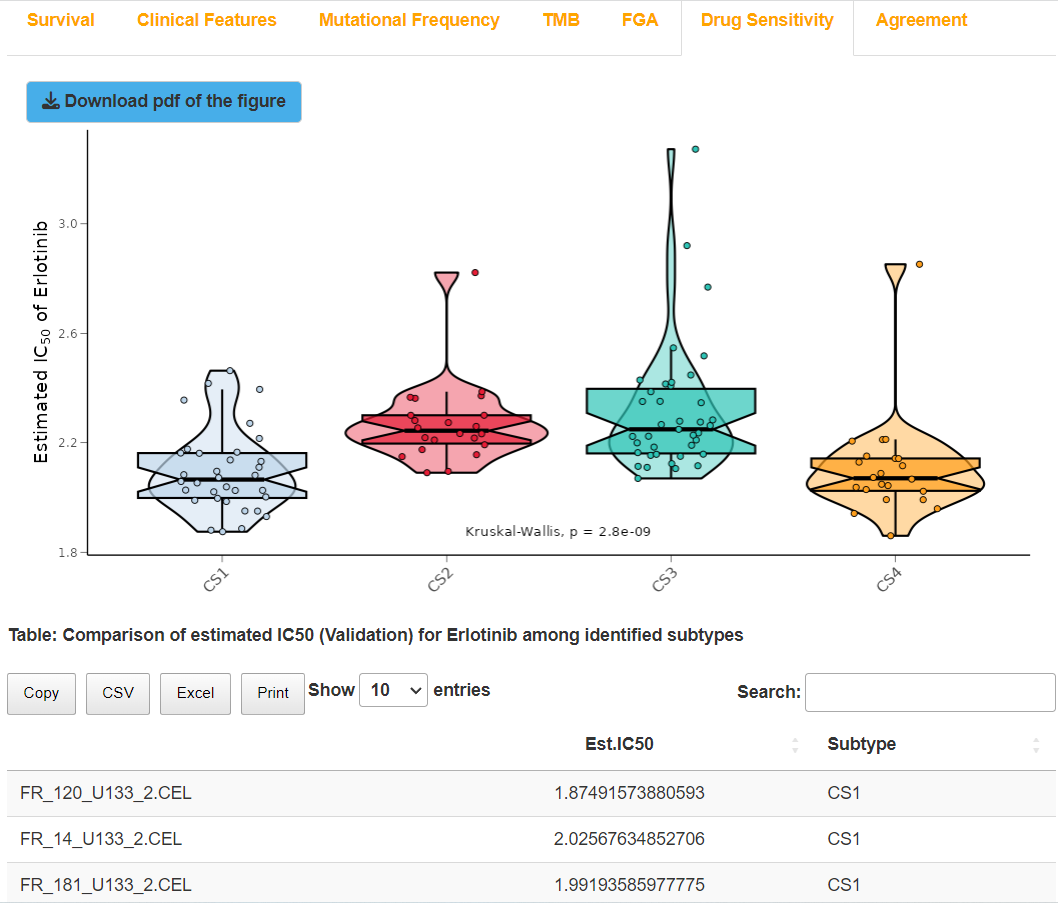


**Figure S55.** Drug sensitivity comparison among subtypes derived from PAM method for Erlotinib on “affy” validation dataset. Abbreviations: PAM, partition around medoids.


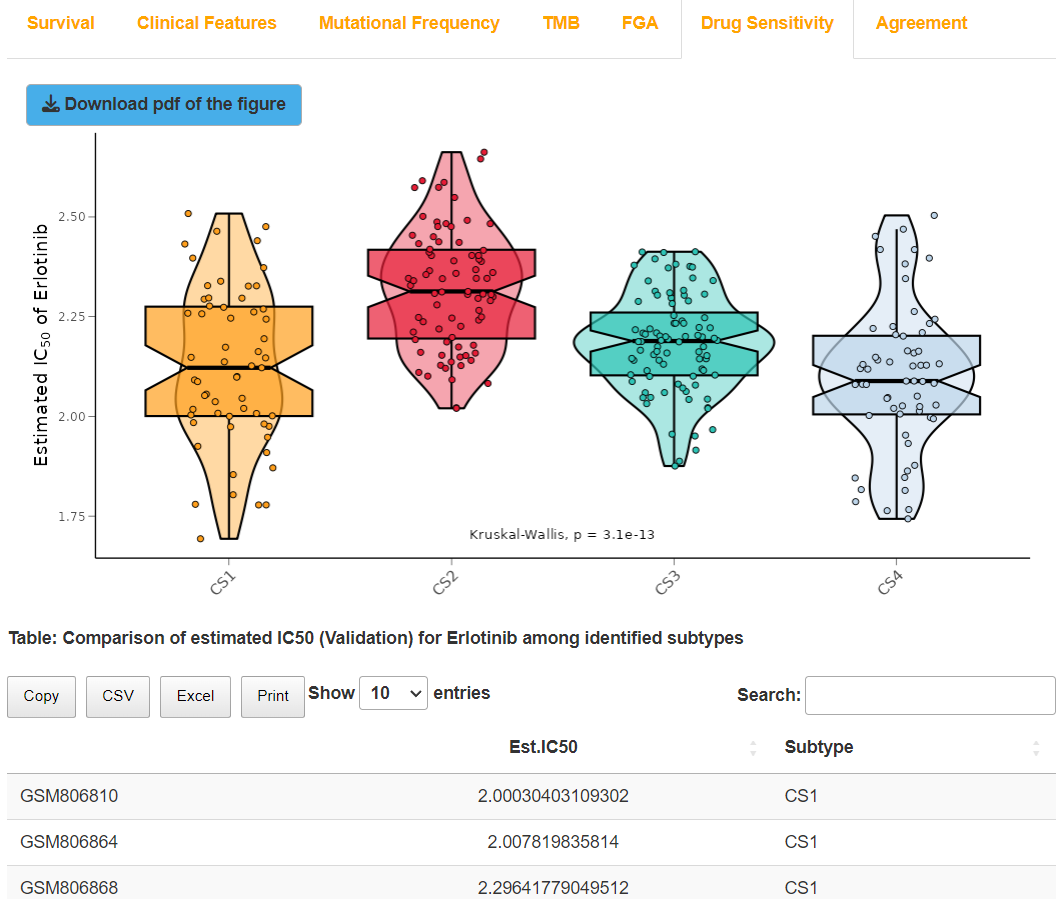


**Figure S56.** Drug sensitivity comparison among subtypes derived from NTP method for Erlotinib on “illumina” validation dataset. Abbreviations: NTP, nearest template prediction.


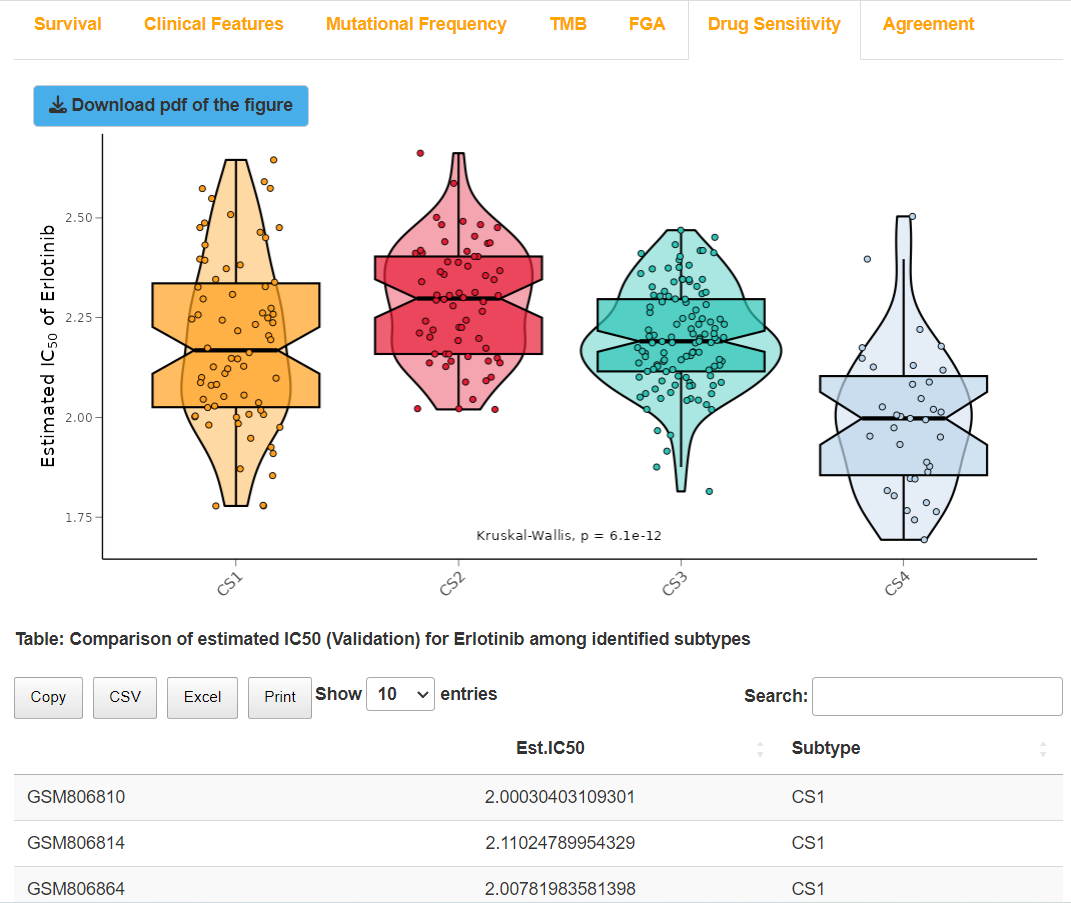


**Figure S57.** Drug sensitivity comparison among subtypes derived from PAM method for Erlotinib on “illumina” validation dataset. Abbreviations: PAM, partition around medoids.


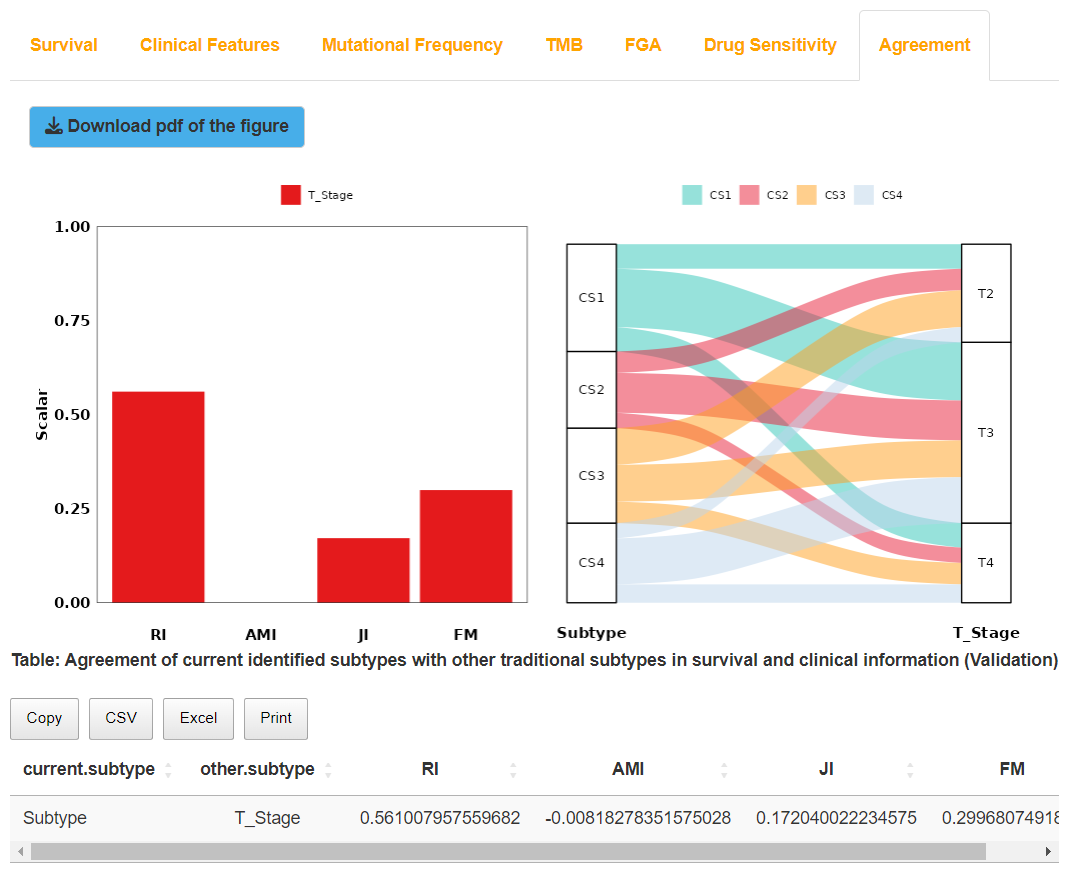


**Figure S58.** Comparison of agreement between other traditional subtypes and subtypes derived from NTP method for “affy” validation dataset. Abbreviations: NTP, nearest template prediction.


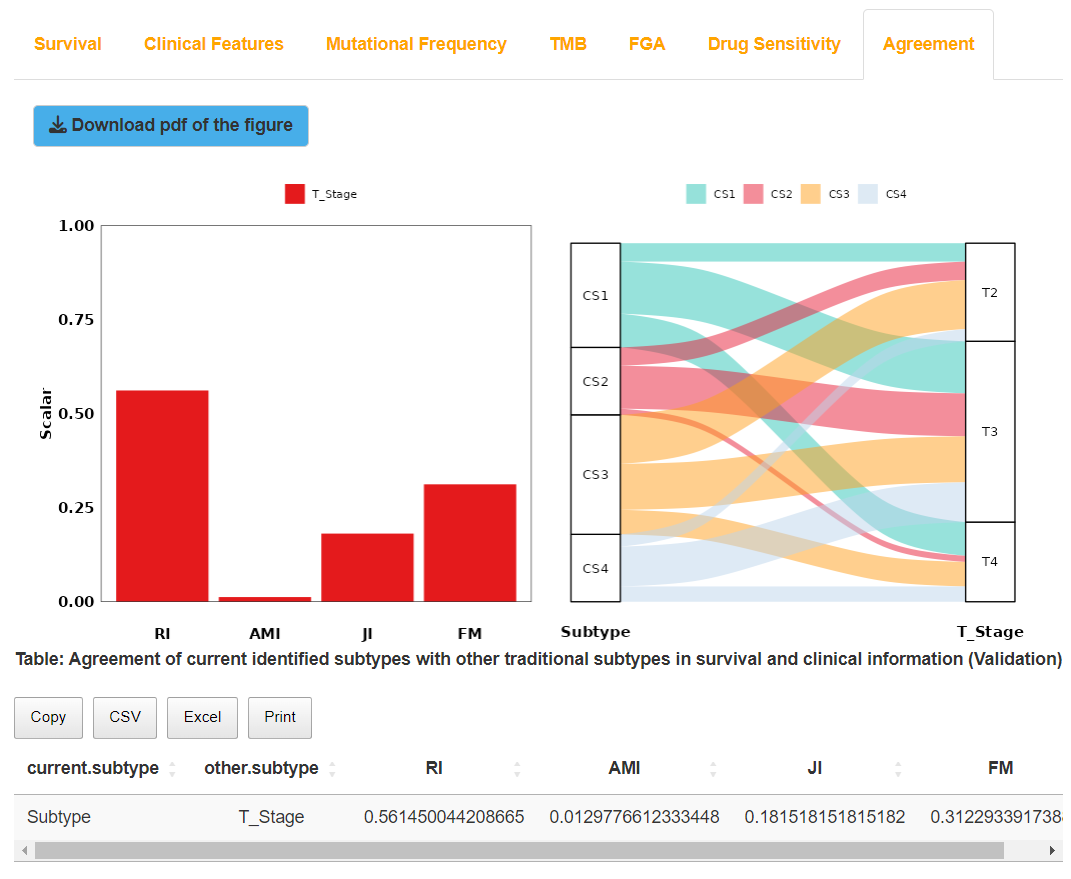


**Figure S59.** Comparison of agreement between other traditional subtypes and subtypes derived from PAM method for “affy” validation dataset. Abbreviations: PAM, partition around medoids.


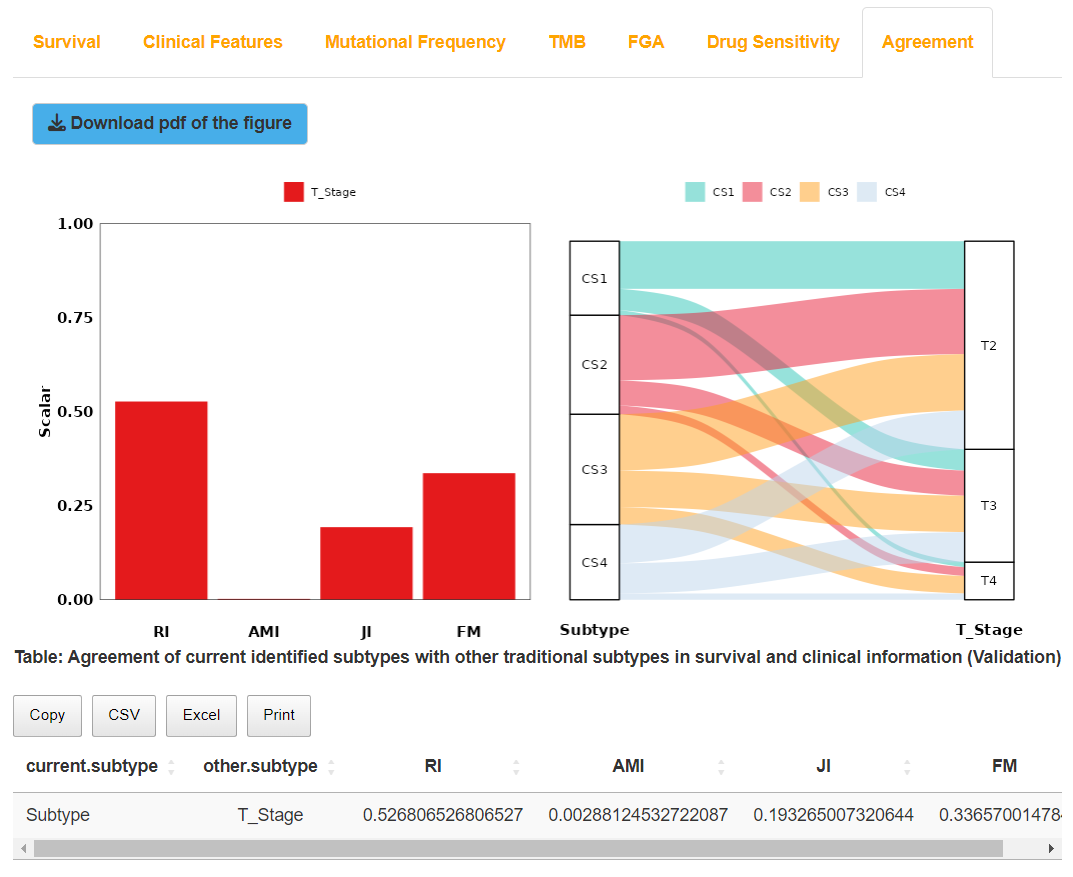


**Figure S60.** Comparison of agreement between other traditional subtypes and subtypes derived from NTP method for “illumina” validation dataset. Abbreviations: NTP, nearest template prediction.


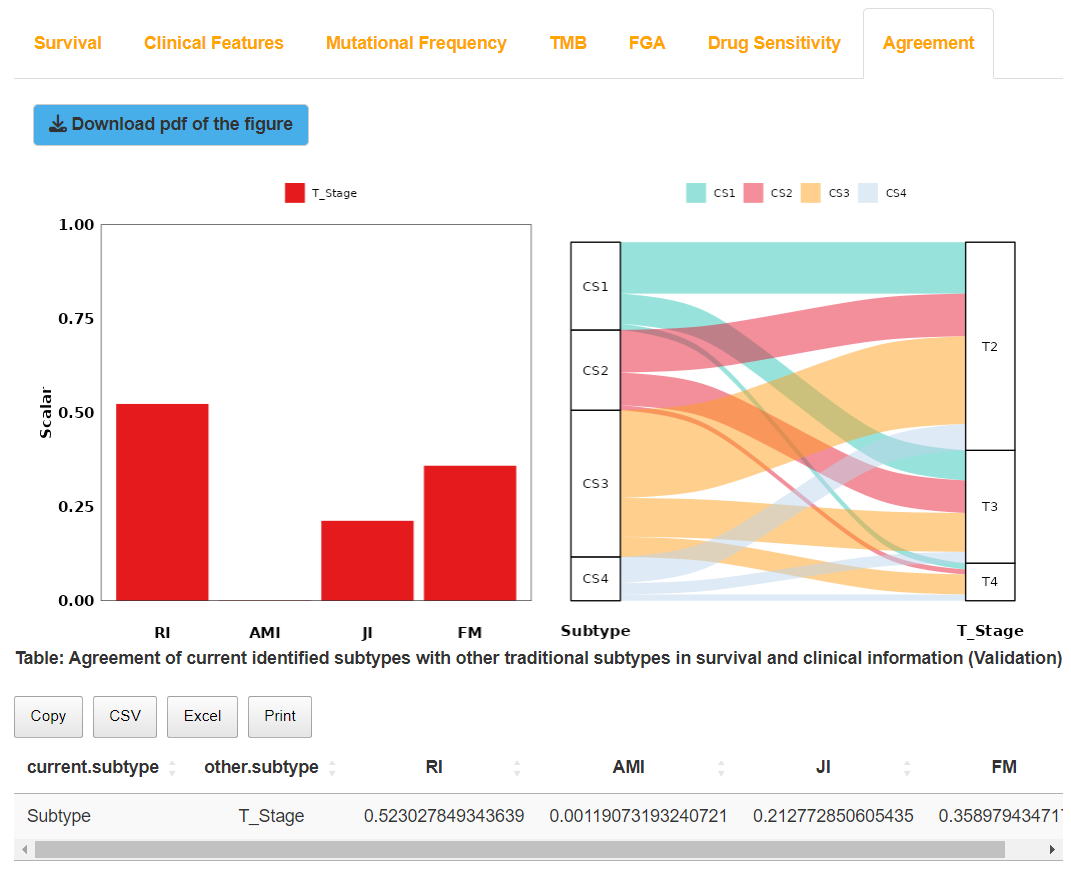


**Figure S61.** Comparison of agreement between other traditional subtypes and subtypes derived from PAM method for “illumina” validation dataset. Abbreviations: PAM, partition around medoids.

# References

1. Kaplan EL, Meier P. Nonparametric estimation from incomplete observations. *Journal of the American statistical association* 1958; **53**: 457-481.

2. Mantel N. Evaluation of survival data and two new rank order statistics arising in its consideration. *Cancer Chemother Rep* 1966; **50**: 163-170.

3. Rand WM. Objective criteria for the evaluation of clustering methods. *Journal of the American Statistical association* 1971; **66**: 846-850.

4. Vinh NX, Epps J, Bailey J. Information theoretic measures for clusterings comparison: is a correction for chance necessary? Proceedings of the 26th annual international conference on machine learning2009. p. 1073-1080.

5. Hancock JM, Bishop MJ. HMMer. *Dictionary of Bioinformatics and Computational Biology* 2004.

6. Fowlkes EB, Mallows CL. A method for comparing two hierarchical clusterings. *Journal of the American statistical association* 1983; **78**: 553-569.

7. Cohen J. A coefficient of agreement for nominal scales. *Educational and psychological measurement* 1960; **20**: 37-46.
